# Comparative effect of statin intensity between prediabetes and type 2 diabetes mellitus after implanting newer-generation drug-eluting stents in Korean acute myocardial infarction patients: a retrospective observational study

**DOI:** 10.1186/s12872-021-02198-w

**Published:** 2021-08-09

**Authors:** Yong Hoon Kim, Ae-Young Her, Myung Ho Jeong, Byeong-Keuk Kim, Sung-Jin Hong, Seunghwan Kim, Chul-Min Ahn, Jung-Sun Kim, Young-Guk Ko, Donghoon Choi, Myeong-Ki Hong, Yangsoo Jang

**Affiliations:** 1 Division of Cardiology, Department of Internal Medicine, Kangwon National University School of Medicine, 156 Baengnyeong Road, 24289 Chuncheon City, Gangwon Province South Korea; 2 Department of Cardiology, Chonnam National University Hospital, Cardiovascular Center, Gwangju, Republic of Korea; 3 Division of Cardiology, Severance Cardiovascular Hospital, Yonsei University College of Medicine, Seoul, Republic of Korea; 4 Division of Cardiology, Inje University College of Medicine, Haeundae Paik Hospital, Busan, Republic of Korea

**Keywords:** Diabetes, Myocardial infarction, Prediabetes, Outcomes, Statin

## Abstract

**Background:**

Comparative studies regarding the long-term clinical outcomes of statin intensity between acute myocardial infarction (AMI) patients with prediabetes and those with type 2 diabetes mellitus (T2DM), after successful implantation of newer-generation drug-eluting stents (DES) with statin treatment, are limited. We compared the 2-year clinical outcomes between these patients.

**Methods:**

A total of 11,612 AMI patients were classified as statin users (n = 9893) and non-users (n = 1719). Thereafter, statin users were further divided into high-intensity (n = 2984) or low-moderate-intensity statin (n = 6909) treatment groups. Those in these two groups were further classified into patients with normoglycemia, prediabetes, and T2DM. The major outcomes were the occurrence of major adverse cardiac events (MACE), defined as all-cause death, recurrent myocardial infarction (Re-MI), or any repeat coronary revascularization.

**Results:**

After adjusting for both high-intensity and low-moderate-intensity statin users, the cumulative incidences of MACE (*p* = 0.737, *p* = 0.062, respectively), all-cause death, Re-MI, and any repeat revascularization were similar between the prediabetes and T2DM groups. In the total study population, both high-intensity and low-moderate-intensity statin treatments showed comparable results. However, in the patients who enrolled after October 2012, the cumulative incidences of MACE (aHR 1.533; 95% CI 1.144–2.053; *p* = 0.004) and any repeat revascularization (aHR, 1.587; 95% CI 1.026–2.456; *p* = 0.038) were significantly lower in high-intensity statin users than in low-moderate intensity statin users. The beneficial effects of high-intensity compared to low-moderate-intensity statin therapy were more apparent in the normoglycemia group than hyperglycemia group, as it reduced the cumulative incidences of MACE (aHR 1.903; 95% CI 1.203–3.010; *p* = 0.006) and any repeat revascularization (aHR 3.248; 95% CI 1.539–6.854; *p* = 0.002).

**Conclusions:**

In this retrospective registry study, prediabetes and T2DM groups showed comparable clinical outcomes, after administering both high-intensity and low-moderate-intensity statin treatments. However, these results are likely to be clearly proved by further studies, especially in patients with AMI who are being treated in contemporary practice.

***Trial registration*:**

Retrospectively registered.

**Supplementary Information:**

The online version contains supplementary material available at 10.1186/s12872-021-02198-w.

## Background

Previous studies showed that high-intensity statin treatment effectively reduced major adverse cardiac events (MACE), cardiac death (CD), recurrent myocardial infarction (Re-MI), and revascularization, in patients with stable angina or acute coronary syndrome [1–4]. Moreover, current guidelines recommend that high-intensity statin treatment should be initiated or continued in all patients with acute MI (AMI), as a class I recommendation [5–8]. However, in many previous studies, the patients were not confined to AMI [2–4], and they received first-generation drug-eluting stents (DES) [2, 9]. Moreover, in actual practice, moderate-dose statin treatment is more commonly administered due to lower bodyweights in Asian population [10]. Prediabetes is not an uncommon population to interventional cardiologists [11]. Recent studies reported that those with prediabetes had worse outcomes compared to normoglycemia and comparable to those with diabetes mellitus (DM) [12, 13]. To reflect contemporary practice in Asian patients and to clarify the different effects of statin-intensity between prediabetes and type 2 DM (T2DM), in patients with AMI, we investigated a two-year clinical outcome in these two groups, especially in Korean AMI patients who underwent successful percutaneous coronary intervention (PCI) using newer-generation DES.

## Methods

### Study design and population

**Fig. 1:**
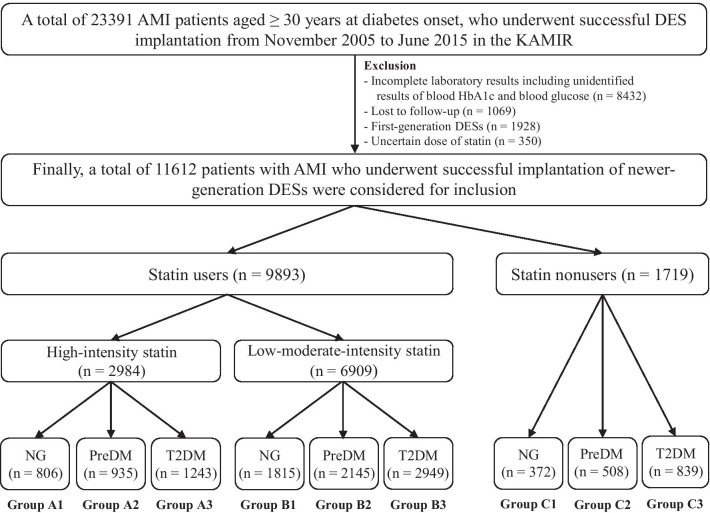
Flowchart showing the patient selection process for the study. AMI: acute myocardial infarction; DES: drug-eluting stents; KAMIR: Korea AMI Registry; NG: normoglycemia; PreDM: prediabetes; T2DM: type 2 diabetes mellitus.

From the Korea AMI Registry (KAMIR) [14], a total of 23,391 AMI patients aged ≥ 30 years at diabetes onset, who underwent successful DES implantation from November 2005 to June 2015, were evaluated. KAMIR [14] is a prospective, observational, and on-line registry with a multicenter cohort study in South Korea, established in November 2005. Details of the registry can be found at the KAMIR website (http://www.kamir.or.kr). In this study, we tried to confine T2DM patients to diabetes cases. Therefore, we defined T2DM based on a previous study [15] which also included patients from the KAMIR. In our study, patients with incomplete laboratory results such as unidentified results of blood hemoglobin (Hb) A1c and blood glucose (n = 8432, 36.1%), patients lost to follow-up (n = 1069, 4.6%), patients treated with first-generation DES (n = 1928, 8.2%), and patients treated with uncertain doses of statins (n = 350, 1.5%) were excluded. Thus, 11,612 AMI patients who received newer-generation DES were included. The types of newer-generation DES used are listed in Table 1. The patients were classified as statin users (n = 9893, 85.2%) and statin non-users (n = 1719, 14.8%). Thereafter, statin users were further divided into high-intensity (n = 2984, 30.2%) and low-moderate-intensity statin users (n = 6909, 69.8%). Finally, those in these two groups (A and B, respectively) were further classified as patients with normoglycemia (group A1 [n = 806, 27.0%] and B1 [n = 1815, 26.3%]), prediabetes (group A2 [n = 935, 31.3%] and B2 [n = 2145, 31.0%]), and T2DM (group A3 [n = 1243, 41.7%] and B3 [n = 2949, 42.7%]) (Fig. 1, Table 1, and Additional file 1: 1). Additionally, over time, patients enrolled later may have benefited from innovative therapies that may have impacted prognosis. To assess how much the results are influenced by this point, we stratified patients into two groups before and after October 2012 according to the enrolled date of individual patient (Additional file 1: 2, 3, 4, and 5). Because a European Society of Cardiology guideline for management of AMI [16] was published in October 2012, and many treatment strategies could be changed according to the newly published guidelines, October 2012 became the cutoff point for our classification. The study protocol was approved by the Institutional Review Board of each participating center, and it was conducted in compliance with the ethical standards of the Declaration of Helsinki 1975. Informed consent was obtained from all patients prior to their inclusion in the study, and we followed up all enrolled patients through face-to-face interviews, phone calls, and chart reviews. All 11,612 patients completed a 2-year clinical follow-up, and all clinical events were evaluated by an independent event adjudication committee. The processes of event adjudication have been described previously by the KAMIR investigators [14].

**Table 1:** Baseline clinical, laboratory, angiographic, and procedural characteristics

	High-intensity statin (Group A, n = 2984)				Low-moderate-intensity statin (Group B, n = 6909)			
	NormoglycemiaGroup A1 (n = 806)	Prediabetes Group A2 (n = 935)	T2DM Group A3 (n = 1243)	*p* value	NormoglycemiaGroup B1 (n = 1815)	Prediabetes Group B2 (n = 2145)	T2DM Group B3 (n = 2949)	*p* value
Age (years)	59.4 ± 12.6	62.0 ± 12.6	62.8 ± 11.4	< 0.001	61.6 ± 12.9	63.6 ± 12.4	64.4 ± 11.7	< 0.001
Male, n (%)	686 (85.1)	736 (78.7)	928 (74.7)	< 0.001	1436 (79.1)	1604 (74.8)	2058 (69.8)	< 0.001
LVEF (%)	54.4 ± 10.3	53.8 ± 10.7	52.1 ± 11.2	< 0.001	52.7 ± 10.2	52.8 ± 10.5	51.3 ± 11.4	< 0.001
BMI (kg/m^2^)	24.3 ± 3.0	24.5 ± 3.2	24.7 ± 3.1	0.042	23.7 ± 2.9	24.1 ± 3.1	24.3 ± 3.1	< 0.001
SBP (mmHg)	134.4 ± 27.1	132.0 ± 28.0	133.0 ± 28.0	0.196	130.8 ± 27.7	129.2 ± 26.4	130.8 ± 27.3	0.070
DBP (mmHg)	83.0 ± 17.0	80.1 ± 16.9	79.8 ± 16.7	< 0.001	80.1 ± 16.2	78.8 ± 15.9	78.9 ± 15.7	0.017
STEMI, n (%)	462 (57.3)	556 (59.5)	681 (54.8)	0.089	1081 (59.6)	1270 (59.2)	1520 (51.5)	< 0.001
Primary PCI, n (%)	451 (97.6)	538 (96.8)	663 (97.4)	0.687	1038 (96.0)	1222 (96.2)	1446 (95.1)	0.315
NSTEMI, n (%)	344 (42.7)	379 (40.5)	562 (45.2)	0.089	734 (40.4)	875 (40.8)	1429 (48.5)	< 0.001
PCI within 24 h	303 (88.1)	337 (88.9)	495 (88.1)	0.913	652 (88.8)	750 (85.7)	1207 (84.5)	0.022
Cardiogenic shock, n (%)	18 (2.2)	33 (3.5)	48 (3.9)	0.120	78 (4.3)	93 (4.3)	126 (4.3)	0.994
Hypertension, n (%)	293 (36.4)	403 (43.1)	705 (56.7)	< 0.001	740 (40.8)	944 (44.0)	1821 (61.7)	< 0.001
Dyslipidemia, n (%)	71 (8.8)	114 (12.2)	161 (13.0)	0.013	163 (9.0)	259 (12.1)	461 (15.6)	< 0.001
Previous MI, n (%)	20 (2.5)	31 (3.3)	50 (4.0)	0.168	59 (3.3)	54 (2.5)	154 (5.2)	< 0.001
Previous PCI, n (%)	30 (3.7)	47 (5.0)	83 (6.7)	0.013	74 (4.1)	105 (4.9)	243 (8.2)	< 0.001
Previous CABG, n (%)	3 (0.4)	1 (0.1)	8 (0.6)	0.145	4 (0.2)	4 (0.2)	25 (0.8)	0.001
Previous HF, n (%)	2 (0.2)	4 (0.4)	13 (1.0)	0.053	10 (0.6)	22 (1.0)	42 (1.4)	0.017
Previous CVA, n (%)	38 (4.7)	42 (4.5)	89 (7.2)	0.011	75 (4.1)	115 (5.4)	241 (8.2)	< 0.001
Current smokers, n (%)	393 (48.8)	473 (50.6)	531 (42.7)	0.001	804 (44.3)	991 (46.2)	1123 (38.1)	< 0.001
Peak CK-MB (mg/dL)	130.9 ± 151.6	142.1 ± 172.4	108.7 ± 151.3	< 0.001	137.6 ± 184.5	145.5 ± 197.4	101.6 ± 135.7	< 0.001
Peak troponin-I (ng/mL)	48.2 ± 75.3	55.0 ± 89.0	47.3 ± 91.7	0.287	48.7 ± 74.7	45.4 ± 83.2	47.6 ± 154.5	0.670
NT-ProBNP (pg/mL)	1466.6 ± 2739.2	1474.5 ± 2226.2	1997.4 ± 4332.4	< 0.001	1570.2 ± 3125.3	1456.4 ± 2160.4	2475.3 ± 6131.6	< 0.001
hs-CRP (mg/dL)	5.39 ± 10.7	5.84 ± 13.3	6.18 ± 18.8	0.522	7.32 ± 28.7	10.2 ± 58.6	10.9 ± 45.2	0.033
Serum creatinine (mg/L)	1.02 ± 1.23	0.98 ± 0.69	1.11 ± 1.02	0.009	0.99 ± 0.82	1.00 ± 0.85	1.21 ± 1.90	< 0.001
eGFR (mL/min/1.73m2)	93.6 ± 46.9	88.6 ± 28.1	88.2 ± 49.0	0.014	92.1 ± 34.2	91.8 ± 44.4	84.0 ± 37.8	< 0.001
Blood glucose (mg/dL)	135.1 ± 39.5	145.9 ± 45.0	227.5 ± 103.4	< 0.001	135.6 ± 48.0	146.7 ± 49.0	223.3 ± 97.8	< 0.001
Hemoglobin A1C (%)	5.4 ± 0.4	6.0 ± 0.2	7.8 ± 1.7	< 0.001	5.3 ± 0.4	6.0 ± 0.2	7.8 ± 3.0	< 0.001
Total cholesterol (mg/dL)	189.9 ± 40.2	199.9 ± 44.3	189.2 ± 53.9	< 0.001	179.2 ± 39.5	186.2 ± 40.9	176.7 ± 45.0	< 0.001
Triglyceride (mg/L)	126.6 ± 84.9	151.2 ± 136.2	157.4 ± 132.6	< 0.001	115.9 ± 89.2	127.1 ± 90.0	147.6 ± 123.1	< 0.001
HDL-cholesterol (mg/L)	44.7 ± 12.1	44.2 ± 18.6	42.1 ± 11.7	< 0.001	44.4 ± 15.5	43.5 ± 13.2	41.8 ± 13.5	< 0.001
LDL-cholesterol (mg/L)	123.3 ± 36.8	130.6 ± 38.9	119.2 ± 40.6	< 0.001	112.9 ± 34.5	119.4 ± 50.7	108.2 ± 35.9	< 0.001
*Discharge medications*								
Aspirin, n (%)	802 (99.6)	931 (99.6)	1241 (99.8)	0.686	1806 (99.5)	2136 (99.6)	2927 (99.3)	0.273
Clopidogrel, n (%)	607 (75.3)	764 (81.7)	1000 (80.5)	0.002	1465 (80.7)	1832 (85.4)	2548 (86.4)	< 0.001
Ticagrelor, n (%)	144 (17.9)	118 (12.6)	159 (12.8)	< 0.001	214 (11.8)	188 (8.8)	234 (7.9)	< 0.001
Prasugrel, n (%)	51 (6.3)	49 (5.2)	82 (6.6)	0.404	127 (7.0)	116 (5.4)	145 (4.9)	0.009
Cilostazole, n (%)	113 (14.0)	168 (18.0)	225 (18.1)	0.034	248 (13.7)	420 (19.6)	611 (20.7)	< 0.001
BBs, n (%)	682 (84.6)	811 (86.7)	1085 (87.3)	0.211	1570 (86.5)	1845 (86.0)	2572 (87.2)	0.449
ACEIs, n (%)	419 (52.0)	470 (50.3)	579 (46.6)	0.042	1161 (64.0)	1344 (62.7)	1669 (56.6)	< 0.001
ARBs, n (%)	239 (29.7)	305 (32.6)	433 (34.8)	0.050	397 (21.9)	463 (21.6)	856 (29.0)	< 0.001
CCBs, n (%)	45 (5.6)	32 (3.4)	78 (6.3)	0.010	93 (5.1)	135 (6.3)	235 (8.0)	< 0.001
*Statin, n (%)*								
Atorvastatin, n (%)	440 (54.6)	418 (44.7)	626 (50.4)	< 0.001	835 (46.0)	987 (46.0)	1475 (50.0)	0.004
Rosuvastatin, n (%)	312 (38.7)	440 (47.1)	501 (40.3)	< 0.001	704 (38.8)	825 (38.5)	967 (32.8)	< 0.001
Simvastatin, n (%)	6 (0.7)	7 (0.7)	8 (0.6)	0.946	152 (8.4)	162 (7.6)	230 (7.8)	0.620
Pitavastatin, n (%)	30 (3.7)	56 (6.0)	75 (6.0)	0.048	98 (5.4)	147 (6.9)	237 (8.0)	0.002
Pravastatin, n (%)	9 (1.1)	8 (0.9)	19 (1.5)	0.350	25 (1.4)	24 (1.1)	39 (1.3)	0.733
Fluvastatin, n (%)	9 (1.1)	6 (0.6)	14 (1.1)	0.462	1 (0.1)	0 (0.0)	1 (0.0)	0.584
*Diabetes management*								
Diet, n (%)			120 (9.7)				221 (7.5)	
Oral agent, n (%)			729 (58.6)				1873 (63.5)	
Insulin, n (%)			64 (5.1)				167 (5.7)	
Untreated, n (%)	-		330 (26.5)				688 (23.3)	
*IRA*								
Left main, n (%)	22 (2.7)	15 (1.6)	31 (2.5)	0.234	25 (1.4)	30 (1.4)	50 (1.7)	0.587
LAD, n (%)	392 (48.6)	453 (48.4)	588 (47.3)	0.777	938 (51.7)	1072 (50.0)	1338 (45.4)	< 0.001
LCx, n (%)	146 (18.1)	156 (16.7)	191 (15.4)	0.259	290 (16.0)	360 (16.8)	504 (17.1)	0.602
RCA, n (%)	246 (30.5)	311 (33.3)	433 (34.8)	0.128	562 (31.0)	683 (31.8)	1057 (35.8)	0.001
*Treated vessel*								
Left main, n (%)	28 (3.5)	27 (2.9)	45 (3.6)	0.626	43 (2.4)	55 (2.6)	84 (2.8)	0.587
LAD, n (%)	471 (58.4)	545 (58.3)	744 (59.9)	0.713	1092 (60.2)	1275 (59.4)	1716 (58.2)	0.374
LCx, n (%)	216 (26.8)	256 (27.4)	365 (29.4)	0.387	443 (26.4)	552 (25.7)	805 (27.3)	0.081
RCA, n (%)	296 (36.7)	373 (39.9)	539 (43.4)	0.010	659 (36.3)	827 (38.6)	1278 (43.3)	< 0.001
*ACC/AHA lesion type*								
Type B1, n (%)	98 (12.2)	115 (12.3)	140 (11.3)	0.717	231 (12.7)	282 (13.1)	363 (12.3)	0.673
Type B2, n (%)	272 (33.7)	292 (31.2)	415 (33.4)	0.457	640 (35.3)	699 (32.6)	987 (33.5)	0.198
Type C, n (%)	365 (45.3)	431 (46.1)	587 (47.2)	0.679	837 (46.1)	974 (45.4)	1373 (46.6)	0.718
*Extent of CAD*								
Single-vessel, n (%)	449 (55.7)	524 (56.0)	569 (45.8)	< 0.001	1003 (55.3)	1106 (51.6)	1244 (42.2)	< 0.001
Two-vessel, n (%)	231 (28.7)	258 (27.6)	428 (34.4)	0.001	546 (30.1)	679 (31.7)	975 (33.1)	0.098
≥ Three-vessel, n (%)	121 (15.0)	150 (16.0)	242 (19.5)	0.018	266 (14.7)	360 (16.8)	730 (24.8)	< 0.001
IVUS, n (%)	209 (25.9)	265 (28.3)	328 (26.4)	0.463	348 (19.2)	496 (23.1)	584 (19.8)	0.003
OCT, n (%)	10 (1.2)	6 (0.6)	9 (0.7)	0.333	11 (0.6)	22 (1.0)	22 (0.7)	0.308
FFR, n (%)	8 (1.0)	15 (1.6)	17 (1.4)	0.539	20 (1.1)	30 (1.4)	43 (1.5)	0.566
*Drug-eluting stents*^a^								
ZES, n (%)	261 (32.4)	321 (34.3)	433 (34.8)	0.504	541 (29.8)	739 (34.5)	993 (33.7)	0.004
EES, n (%)	426 (52.9)	498 (53.3)	649 (52.2)	0.885	944 (52.0)	1091 (50.9)	1539 (52.2)	0.622
BES, n (%)	135 (16.7)	128 (13.7)	175 (14.1)	0.146	322 (17.7)	307 (14.3)	384 (13.0)	< 0.001
Others, n (%)	5 (0.6)	10 (1.1)	18 (1.4)	0.214	46 (2.5)	55 (2.6)	89 (3.0)	0.500
Stent diameter (mm)	3.17 ± 0.42	3.16 ± 0.42	3.14 ± 0.43	0.235	3.15 ± 0.42	3.13 ± 0.41	3.09 ± 0.41	< 0.001
Stent length (mm)	27.7 ± 11.2	27.6 ± 12.7	28.0 ± 12.5	0.681	27.4 ± 11.8	27.0 ± 11.1	27.8 ± 11.9	0.051
Number of stent	1.48 ± 0.80	1.51 ± 0.84	1.59 ± 0.87	0.007	1.40 ± 0.72	1.47 ± 0.78	1.54 ± 0.82	< 0.001

Values are means ± SD or numbers and percentages. The *p* values for continuous data obtained from the analysis of variance. The *p* values for categorical data from chi-square or Fisher’s exact test. LVEF: left ventricular ejection fraction; BMI: body mass index; SBP: systolic blood pressure; DBP: diastolic blood pressure; STEMI: ST-elevation myocardial infarction; NSTEMI: non-ST-elevation myocardial infarction; PCI: percutaneous coronary intervention; CABG: coronary artery bypass graft; HF: heart failure; CVA: cerebrovascular accident; CK-MB: creatine kinase myocardial band; NT-ProBNP: N-terminal pro-brain natriuretic peptide; hs-CRP: high sensitivity C-reactive protein; eGFR: estimated glomerular filtration rate; HDL: high-density lipoprotein; LDL: low-density lipoprotein; BBs: beta-blockers; ACEs: angiotensin converting enzyme inhibitors; ARBs: angiotensin receptor blockers; CCBs: calcium channel blockers; IRA: infarct-related artery; LAD: left anterior descending coronary artery; LCx: left circumflex coronary artery; RCA: right coronary artery; ACC/AHA: American College of Cardiology/American Heart Association; CAD: coronary artery disease; IVUS: intravascular ultrasound; OCT: optical coherence tomography; FFR: fractional flow reserve; ZES: zotarolimus-eluting stent; EES: everolimus-eluting stent; BES: biolimus-eluting stents

^a^Drug-eluting stents were composed of ZES (Resolute Integrity stent; Medtronic, Inc., Minneapolis, MN), EES (Xience Prime stent, Abbott Vascular, Santa Clara, CA; or Promus Element stent, Boston Scientific, Natick, MA), BES (BioMatrix Flex stent, Biosensors International, Morges, Switzerland; or Nobori stent, Terumo Corporation, Tokyo, Japan), and others include any other newer-generation drug-eluting stents except for ZES, EES, and BES

### Percutaneous coronary intervention (PCI) procedure and medical treatment

Diagnostic coronary angiography and PCI were performed using standard techniques [17]. All patients received loading doses of aspirin (200–300 mg) and other antiplatelet agents such as clopidogrel (300–600 mg), ticagrelor (180 mg), or prasugrel (60 mg), before PCI was performed. It was recommended that the duration of dual antiplatelet therapy (DAPT; a combination of aspirin 100 mg/day with clopidogrel 75 mg/day, ticagrelor 90 mg twice daily, or prasugrel 5–10 mg/day) should be for at least 1 year after the index PCI. Based on previous reports [18, 19], triple antiplatelet therapy (TAPT; cilostazol [100 mg twice daily] combined with DAPT) was determined by the individual operator’s discretion. In this study, the patients who received atorvastatin, rosuvastatin, simvastatin, pitavastatin, pravastatin, and fluvastatin were included (Table 1), and the kind and dose of statins to be used was left at the physicians’ discretion.

### Study definitions and clinical outcomes

In this study, as mentioned [10], because moderate-dose statin treatment is more commonly administered due to lower bodyweights in Asian population, atorvastatin (≥ 40 mg), rosuvastatin (≥ 20 mg), simvastatin (≥ 40 mg), pitavastatin (≥ 4 mg), and pravastatin (≥ 40 mg) were considered as high-intensity statins, while others were considered as low-moderate-intensity statins [20], compared with current guideline [21]. Glycemic status was determined by the clinical practice recommendations of the American Diabetes Association [22]. T2DM was defined as either known T2DM for which patients received medical treatment (insulin or antidiabetics) or newly diagnosed T2DM defined as an HbA1c level ≥ 6.5%, a fasting plasma glucose (FPG) level ≥ 126 mg/dL (7 mmol/L), and/or random plasma glucose (RPG) level ≥ 200 mg/dL (11.1 mmol/L), during the index hospitalization or according to their medical history. Prediabetes was defined as an HbA1c level of 5.7%–6.4% and an FPG of 100–125 mg/dL (5.6–6.9 mmol/L). Moreover, in the case of discrepancies between HbA1c and FPG or RPG levels, we made HbA1c level a priority [12]. AMI was defined according to the current guidelines [5–8]. Estimated glomerular filtration rate (eGFR) was calculated using the Modification of Diet in Renal Disease (MDRD) study equation [23]. The major outcome was the occurrence of MACE defined as all-cause death, Re-MI, or any repeat coronary revascularization. All-cause death was classified as CD or non-CD. Any repeat revascularization comprised of target lesion revascularization (TLR), target vessel revascularization (TVR), and non-TVR. The definitions of Re-MI, TLR, TVR, and non-TVR have been published previously [24].

### Statistical analysis

The normality test was conducted using the Kolmogorov–Smirnov test. Categorical data were reported as numbers and percentages, and they were compared using the chi-square or Fisher’s exact test, as appropriate. For continuous variables, differences among the three groups are evaluated using an analysis of variance or the Jonckheere-Terpstra test, while a post-hoc analysis was performed using the Hochberg test or Dunnett T3 test. The data were expressed as mean ± standard deviation. To determine meaningful variables, all variables with *p* < 0.001 were included in the univariate analysis (Additional file 1: 6). After univariate analysis, variables with *p* < 0.001 and known conventional risk factors of poor outcomes in the AMI population were considered potential confounding factors, and were entered into the multivariate analysis [25]. Various clinical outcomes were estimated using the Kaplan–Meier method, and intergroup differences were compared using the log-rank test. For all analyses, a two-sided *p* value < 0.05 was considered statistically significant. All statistical analyses were performed using the SPSS software version 20 (IBM, Armonk, NY, USA).

## Results

### Baseline characteristics

Table 1, Additional file 1: 1, 7, 8, and 9 show the baseline characteristics of the study population. Both in high-intensity (group A) and in low-moderate-intensity (group B) statin users, the number of men, single-vessel disease, and the prescription rates of ticagrelor and angiotensin-converting enzyme inhibitors (ACEIs) were the highest in normoglycemia groups (group A1 and B1). The number of current smokers and peak creatine kinase-MB level and the levels of total and low-density lipoprotein (LDL) cholesterols were the highest in prediabetes groups (group A2 and B2). The mean age and the number of patents with hypertension, dyslipidemia, previous history of PCI and cerebrovascular accidents; levels of N-terminal pro-brain natriuretic peptide, serum creatinine, and triglyceride; the prescription rates of cilostazole and calcium channel blockers; the number of cases with right coronary artery (RCA) as infarc-related artery (IRA) and treated vessel, multivessel disease, and deployed stents, were the highest in T2DM group (group A3 and B3).

### Clinical outcomes

**Fig. 2:**
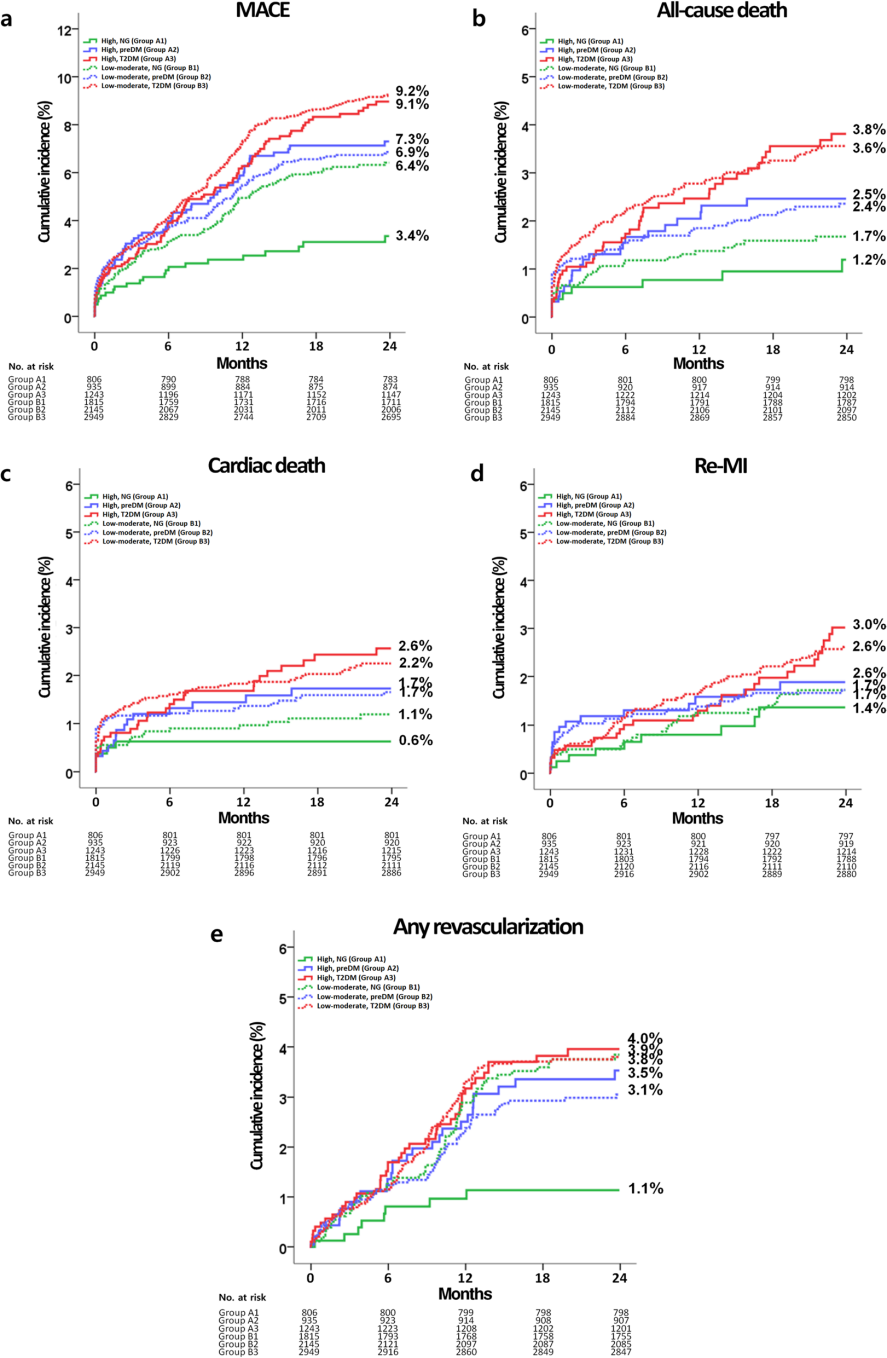
Kaplan–Meier analyses for the MACE (**a**), all-cause death (**b**), cardiac death (**c**), Re-MI (**d**), any repeat revascularization (**e**) in statin users. MACE: major adverse cardiac events; Re-MI: recurrent myocardial infarction, high: high-intensity statin; low-moderate: low-moderate-intensity statin; NG: normoglycemia; preDM: prediabetes; T2DM: type 2 diabetes mellitus

In both high-intensity and low-moderate intensity statin users, the comparisons of clinical outcomes among the three glycemic groups during the 2-year follow-up period are presented in Tables 2, 3, and Fig. 2. In high-intensity statin users, the cumulative incidences of MACE (adjusted hazard ratio [aHR]: 2.187; 95% confidence interval [CI]: 1.341–3.569; *p* = 0.002) and any repeat revascularization (aHR 3.009; 95% CI 1.342–6.745; *p* = 0.006) were higher in group A2 (prediabetes) than in group A1 (normoglycemia). Similarly, the cumulative incidences of MACE (aHR 2.368; 95% CI 1.480–3.788; *p* = 0.001) and any repeat revascularization (aHR 3.619; 95% CI 1.659–7.898; *p* = 0.001) were significantly higher in group A3 (T2DM) than in group A1. However, the cumulative incidences of MACE, all-cause death, CD, Re-MI, and any repeat revascularization were similar between groups A2 and A3 (Table 2). In low-moderate-intensity statin users, the cumulative incidences of MACE, all-cause death, CD, Re-MI, and any repeat revascularization were not significantly different between groups B1 (normoglycemia) and B2 (prediabetes) as well as between groups B2 and B3 (T2DM). However, the cumulative incidences of MACE (aHR 1.285; 95% CI 1.014–1.629; *p* = 0.038) and all-cause death (aHR 1.784; 95% CI 1.156–2.751; *p* = 0.009) were significantly higher in group B3 than in group B1. In normoglycemia groups (Table 3), the cumulative incidences of MACE (aHR 1.903; 95% CI 1.203–3.010; *p* = 0.006) and any repeat revascularization (aHR 3.248; 95% CI 1.539–6.854; *p* = 0.002) were significantly lower in high-intensity than in low-moderate-intensity statin users. However, both in the prediabetes (groups A2 and B2) and T2DM (group A3 and B3) groups, the cumulative incidences of MACE, all-cause death, CD, Re-MI, and any repeat revascularization were similar between high-intensity (group A2 vs. B2) and low-moderate-intensity statin users (group A3 vs. B3). Furthermore, in the total study population, there were no significant differences in major clinical outcomes between high-intensity (A1 + A2 + A3) and low-moderate-intensity stain users (B1 + B2 + B3). In high-intensity statin users, in both before and after October 2012 groups (Additional file 1: 2 and 3), the cumulative incidences of MACE (aHR 2.635; *p* = 0.003 and aHR 1.845; *p* = 0.048, respectively) and any repeat revascularization (aHR 4.162; *p* = 0.002 and aHR 2.845; *p* = 0.044, respectively) were higher in group A2 than in group A1. The cumulative incidences of MACE (aHR 2.896; *p* = 0.002 and aHR 2.146; *p* = 0.033, respectively) and any repeat revascularization (aHR 4.666; *p* = 0.001and aHR 3.241; *p* = 0.040, respectively) were significantly higher in group A3 than in group A1. In low-moderate-intensity statin users, in before October 2012 group (Additional file 1: 2), the cumulative incidence of all-cause death was significantly higher in group B3 than in group B1 (aHR 1.621; 95% CI 1.102–2.614; *p* = 0.044). In after October 2012 group (Additional file 1: 3), the cumulative incidences of MACE (aHR 1.429; 95% CI 1.001–1.998; *p* = 0.043), all-cause death (aHR 2.940; 95% CI 1.388–6.225; *p* = 0.005), CD (aHR 3.319; 95% CI 1.235–8.919; *p* = 0.017) were significantly higher in group B3 than in group B1. Moreover, the cumulative incidence of CD (aHR 2.757; 95% CI 1.038–7.327; *p* = 0.042) was significantly higher in group B3 than in group B2. In before October 2012 group (Additional file 1: 4), in normoglycemia groups, the cumulative incidences of any repeat revascularization (aHR 3.025; 95% CI 1.045–8.760; *p* = 0.041) was significantly lower in high-intensity than in low-moderate-intensity statin users In after October 2012 group (Additional file 1: 5), in both normoglycemia groups and total study population, the cumulative incidences of MACE (aHR 2.002; *p* = 0.042 and aHR 1.533; *p* = 0.004, respectively) and any repeat revascularization (aHR 3.308; *p* = 0.028 and aHR 1.587; *p* = 0.038, respectively) were significantly lower in high-intensity than in low-moderate-intensity statin users. In the comparison of major clinical outcomes between statin users and non-users (Additional file 1: 10), statin non-users showed higher cumulative incidences of MACE, all-cause death, and CD in all three glycemic statuses. Additionally, in the T2DM group, the cumulative incidence of any repeat revascularization (aHR 1.637; 95% CI 1.171–32.290; *p* = 0.004) was significantly higher in statin non-users than in statin users. Moreover, in the total study population, the cumulative incidences of MACE, all-cause death, CD, and any repeat revascularization were significantly higher in statin non-users than in statin users.

**Table 2:** Clinical outcomes in high-intensity or low-moderate-intensity statin users at 2 years

	Group A1 Normoglycemia	Group A2 Prediabetes	Log-Rank	Unadjusted		Adjusted^a^	
				HR (95% CI)	*p* value	HR (95% CI)	*p* value
*High-intensity statin*							
MACE	23 (3.4)	61 (7.3)	0.001	2.262 (1.400–3.655)	0.001	2.187 (1.341–3.569)	0.002
All-cause death	8 (1.2)	21 (2.5)	0.049	2.218 (0.983–5.009)	0.055	2.155 (0.935–4.967)	0.071
Cardiac death	5 (0.6)	15 (1.7)	0.058	2.570 (0.934–7.072)	0.068	2.687 (0.929–7.768)	0.067
Re-MI	9 (1.4)	16 (1.9)	0.325	1.503 (0.664–3.402)	0.328	1.390 (0.606–3.189)	0.438
Any repeat revascularization	8 (1.1)	28 (3.5)	0.005	2.958 (1.348–6.490)	0.007	3.009 (1.342–6.745)	0.006

	Group A1 Normoglycemia	Group A3 T2DM	Log-Rank	Unadjusted		Adjusted^a^	
				HR (95% CI)	*p* value	HR (95% CI)	*p* value
*High-intensity statin*							
MACE	23 (3.4)	96 (9.0)	< 0.001	2.651 (1.682–4.179)	< 0.001	2.368 (1.480–3.788)	0.001
All-cause death	8 (1.2)	41 (3.8)	< 0.001	3.215 (1.507–6.859)	0.003	2.244 (1.035–4.866)	0.051
Cardiac death	5 (0.6)	28 (2.6)	0.005	3.540 (1.367–9.169)	0.009	2.474 (0.932–6.566)	0.069
Re-MI	9 (1.4)	29 (3.0)	0.063	2.004 (0.948–4.233)	0.069	2.051 (0.943–4.460)	0.070
Any repeat revascularization	8 (1.1)	42 (4.0)	0.001	3.330 (1.563–7.093)	0.002	3.619 (1.659–7.898)	0.001

	Group A2 Prediabetes	Group A3 T2DM	Log-Rank	Unadjusted		Adjusted^a^	
				HR (95% CI)	*p* value	HR (95% CI)	*p* value
*High-intensity statin*							
MACE	61 (7.3)	96 (9.0)	0.316	1.178 (0.855–1.624)	0.316	1.059 (0.760–1.474)	0.737
All-cause death	21 (2.5)	41 (3.8)	0.151	1.467 (0.867–2.482)	0.153	1.181 (0.686–2.033)	0.547
Cardiac death	15 (1.7)	28 (2.6)	0.291	1.399 (0.747–2.620)	0.294	1.063 (0.554–2.039)	0.854
Re-MI	16 (1.9)	29 (3.0)	0.307	1.373 (0.746–2.527)	0.309	1.384 (0.719–2.529)	0.352
Any repeat revascularization	28 (3.5)	42 (4.0)	0.628	1.125 (0.698–1.815)	0.628	1.042 (0.637–1.706)	0.869

	Group B1 Normoglycemia	Group B2 Prediabetes	Log-rank	Unadjusted		Adjusted^b^	
				HR (95% CI)	*p* value	HR (95% CI)	*p* value
*Low-moderate-intensity statin*							
MACE	104 (6.4)	139 (6.9)	0.509	1.089 (0.845–1.405)	0.509	1.076 (0.831–1.393)	0.579
All-cause death	28 (1.7)	48 (2.4)	0.150	1.406 (0.882–2.240)	0.152	1.289 (0.803–2.071)	0.293
Cardiac death	20 (1.1)	34 (1.7)	0.227	1.403 (0.807–2.437)	0.230	1.205 (0.687–2.114)	0.516
Re-MI	27 (1.7)	35 (1.7)	0.819	1.060 (0.642–1.752)	0.819	1.414 (0.684–1.902)	0.613
Any repeat revascularization	60 (3.9)	60 (3.1)	0.229	1.245 (0.870–1.781)	0.230	1.219 (0.846–1.755)	0.288

	Group B1 Normoglycemia	Group B3 T2DM	Log-rank	Unadjusted		Adjusted^b^	
				HR (95% CI)	*p* value	HR (95% CI)	*p* value
*Low-moderate-intensity statin*							
MACE	104 (6.4)	254 (9.2)	0.001	1.463 (1.164–1.837)	0.001	1.285 (1.014–1.629)	0.038
All-cause death	28 (1.7)	99 (3.6)	< 0.001	2.124 (1.396–3.231)	< 0.001	1.784 (1.156–2.751)	0.009
Cardiac death	20 (1.1)	63 (2.2)	0.011	1.899 (1.148–3.141)	0.012	1.527 (0.906–2.572)	0.112
Re-MI	27 (1.7)	69 (2.6)	0.065	1.517 (0.972–2.367)	0.067	1.422 (0.894–2.260)	0.137
Any repeat revascularization	60 (3.9)	102 (3.8)	0.938	1.013 (0.736–1.393)	0.938	1.085 (0.778–1.515)	0.630

	Group B2 Prediabetes	Group B3 T2DM	Log-rank	Unadjusted		Adjusted^b^	
				HR (95% CI)	*p* value	HR (95% CI)	*p* value
*Low-moderate-intensity statin*							
MACE	139 (6.9)	254 (9.2)	0.005	1.347 (1.096–1.657)	0.005	1.241 (1.004–1.534)	0.062
All-cause death	48 (2.4)	99 (3.6)	0.018	1.512 (1.071–2.135)	0.019	1.383 (0.970–1.970)	0.083
Cardiac death	34 (1.7)	63 (2.2)	0.151	1.355 (0.893–2.056)	0.153	1.203 (0.784–1.848)	0.397
Re-MI	35 (1.7)	69 (2.6)	0.072	1.449 (0.965–2.176)	0.074	1.331 (0.878–2.019)	0.178
Any repeat revascularization	60 (3.1)	102 (3.8)	0.157	1.258 (0.915–1.731)	0.158	1.165 (0.841–1.615)	0.358

HR: Hazard ratio; CI: confidence interval; T2DM: type 2 diabetes mellitus; MACE: major adverse cardiac events; Re-MI: recurrent myocardial infarction; LVEF: left ventricular ejection fraction; STEMI: ST-segment elevation myocardial infarction; CVA: cerebrovascular accidents; NT-ProBNP: N-terminal pro-brain natriuretic peptide; eGFR: estimated glomerular filtration rate; ACC/AHA: American College of Cardiology/American Heart Association; ACEI: angiotensin-converting enzyme inhibitors; ARB: angiotensin receptor blockers

^a^Adjusted by age, male, LVEF, cardiogenic shock, STEMI, hypertension, previous MI, previous CVA, current smoker, NT-ProBNP, serum creatinine, eGFR, atorvastatin, ACC/AHA type B2 lesion, ≥ Three-vessel disease, and number of stent

^b^Adjusted by age, male, LVEF, cardiogenic shock, STEMI, hypertension, previous MI, previous CVA, current smoker, NT-ProBNP, serum creatinine, eGFR, total cholesterol, beta-blocker, ACEI, ARB, rosuvastatin, simvastatin, intravascular ultrasound, single-vessel disease, ≥ three-vessel disease, and number of stent

**Table 3:** Clinical outcomes between high-intensity and low-moderate-intensity statin in three different glycemic statuses at 2 years

Outcomes	High-intensity (n = 806)	Low-moderate-intensity (n = 1815)	Log-rank	Unadjusted		Adjusted^a^	
				HR (95% CI)	*p* value	HR (95% CI)	*p* value
Normoglycemia	Group A1	Group B1					
MACE	23 (3.4)	104 (6.4)	0.004	1.924 (1.225–3.022)	0.005	1.903 (1.203–3.010)	0.006
All-cause death	8 (1.2)	28 (1.7)	0.311	1.497 (0.682–3.286)	0.314	1.342 (0.604–2.982)	0.471
Cardiac death	5 (0.6)	20 (1.1)	0.265	1.733 (0.650–4.619)	0.271	1.628 (0.602–4.402)	0.336
Re-MI	9 (1.4)	27 (1.7)	0.555	1.254 (0.590–2.668)	0.556	1.238 (0.570–2.690)	0.589
Any revascularization	8 (1.1)	60 (3.9)	0.001	3.146 (1.504–6.579)	0.002	3.248 (1.539–6.854)	0.002

Outcomes	High-intensity (n = 935)	Low-moderate-intensity (n = 2145)	Log-rank	Unadjusted		Adjusted^a^	
				HR (95% CI)	*p* value	HR (95% CI)	*p* value
Prediabetes	Group A2	Group B2					
MACE	61 (7.3)	139 (6.9)	0.656	1.071 (0.792–1.447)	0.656	1.112 (0.818–1.510)	0.499
All-cause death	21 (2.5)	48 (2.4)	0.846	1.052 (0.630–1.757)	0.846	1.103 (0.653–1.862)	0.715
Cardiac death	15 (1.7)	34 (1.7)	0.885	1.046 (0.569–1.920)	0.886	1.014 (0.542–1.897)	0.966
Re-MI	16 (1.9)	35 (1.7)	0.769	1.092 (0.605–1.974)	0.769	1.166 (0.639–2.130)	0.617
Any revascularization	28 (3.5)	60 (3.1)	0.520	1.158 (0.740–1.814)	0.521	1.152 (0.730–1.819)	0.543

Outcomes	High-intensity (n = 1243)	Low-moderate-intensity (n = 2949)	Log-rank	Unadjusted		Adjusted^a^	
				HR (95% CI)	*p* value	HR (95% CI)	*p* value
T2DM	Group A3	Group B3					
MACE	96 (9.0)	254 (9.2)	0.654	1.055 (0.834–1.335)	0.654	1.010 (0.796–1.282)	0.934
All-cause death	41 (3.8)	99 (3.6)	0.876	1.029 (0.715–1.482)	0.876	1.139 (0.787–1.650)	0.491
Cardiac death	28 (2.6)	63 (2.2)	0.690	1.095 (0.701–1.709)	0.690	1.238 (0.788–1.945)	0.354
Re-MI	29 (3.0)	69 (2.6)	0.748	1.074 (0.696–1.657)	0.748	1.108 (0.713–1.720)	0.649
Any revascularization	42 (4.0)	102 (3.8)	0.843	1.037 (0.724–1.486)	0.843	1.059 (0.736–1.526)	0.756

Outcomes	High-intensity (n = 2984)	Low-moderate-intensity (n = 6909)	Log-rank	Unadjusted		Adjusted^a^	
				HR (95% CI)	*p* value	HR (95% CI)	*p* value
Total	Group A1 + A2 + A3	Group B1 + B2 + B3					
MACE	180 (7.0)	497 (7.8)	0.164	1.129 (0.952–1.339)	0.164	1.070 (0.900–1.273)	0.444
All-cause death	70 (2.7)	175 (2.7)	0.824	1.032 (0.782–1.362)	0.825	1.083 (0.816–1.436)	0.581
Cardiac death	48 (1.8)	117 (1.8)	0.920	1.017 (0.727–1.424)	0.920	1.099 (0.781–1.546)	0.590
Re-MI	54 (2.2)	131 (2.1)	0.922	1.016 (0.740–1.395)	0.922	1.066 (0.771–1.473)	0.700
Any revascularization	78 (3.1)	222 (3.6)	0.285	1.151 (0.889–1.490)	0.286	1.131 (0.870–1.470)	0.358

HR: Hazard ratio; CI: confidence interval; T2DM: type 2 diabetes mellitus; MACE: major adverse cardiac events; Re-MI: recurrent myocardial infarction; LVEF: left ventricular ejection fraction; BMI: body mass index; SBP: systolic blood pressure; DBP: diastolic blood pressure; NT-ProBNP: N-terminal pro-brain natriuretic peptide; LDL: low-density lipoprotein; ACEI: angiotensin-converting enzyme inhibitors; ARBs: angiotensin receptor blockers; IVUS: intravascular ultrasound

^a^Adjusted by age, male, LVEF, BMI, SBP, DBP, cardiogenic shock, hypertension, current smoker, NT-ProBNP, total cholesterol, triglyceride, LDL-cholesterol, ticagrelor, ACEI, ARB, atorvastatin, rosuvastatin, simvastatin, IVUS, and stent diameter

Independent predictors for MACE in high-intensity statin users and in low-moderate-intensity statin users at 2 years are listed in Additional file 1: 11 and 12. In both high-intensity and low-moderate-intensity statin users, decreased left ventricular ejection fraction (LVEF) (< 40%), cardiogenic shock, and decreased eGFR (< 60 mL/min/1.73m^2^) were found to be significantly common independent predictors for MACE.

## Discussion

The main findings were as follows: (1) the cumulative incidences of MACE, all-cause death, CD, Re-MI, and any repeat revascularization were similar between the prediabetes and T2DM groups in both high-intensity and low-moderate-intensity statin users; (2) in high-intensity statin users, the cumulative incidences of MACE and any repeat revascularization in both prediabetes and T2DM group were higher than those in the normoglycemia group; (3) in low-moderate-intensity statin users, the cumulative incidences of MACE and all-cause death were significantly higher in T2DM than in normoglycemia group; (4) in both patients who enrolled after October 2012 and normoglycemia group, high-intensity statin treatment was more effective in reducing MACE and any revascularization than low-moderate-intensity statin treatment; (5) in the total population, statin users showed significantly lower incidences of MACE, all-cause death, CD, and any repeat revascularization than non-users did; (6) in both high-intensity and low-moderate-intensity statin users, decreased LVEF, cardiogenic shock, and decreased eGFR were common independent predictors of MACE.

According to current guidelines [8, 21], regardless of glycemic status, early and intensive statin treatment is recommended; more intensive statin treatment greatly reduced the risks of CD, non-fatal MI, and coronary revascularization [4]. Consistent with these previous reports [4, 8, 21], our study showed that the cumulative incidences of MACE, all-cause death, CD, and any repeat revascularization were significantly lower in statin users than in statin non-users in the total study population (Additional file 1: 10). However, with respect to statin intensity, in the total statin users groups, high-intensity and low-moderate-intensity statin users showed comparable clinical outcomes. Our results were similar to those of a previous report, which also included patients from the KAMIR [20]. In that study [20], the risk of MACE was similar between high-intensity and low-moderate-intensity statin users (HR: 0.917; 95% CI 0.760–1.107; *p* = 0.368). A possible explanation for this similarity may be related with different definitions, which confined high-intensity statin treatment to Asian patients, compared with the current guideline [21]. However, in the patients who enrolled after October 2012, the cumulative incidences of MACE (aHR 1.533; 95% CI 1.144–2.053; *p* = 0.004) and any repeat revascularization (aHR, 1.587; 95% CI 1.026–2.456; *p* = 0.038) were significantly lower in high-intensity statin users than in low-moderate intensity statin users. This finding also could reflect the possibility that innovative therapies may have impacted prognosis. Despite this limitation, in the normoglycemia group, the cumulative incidences of MACE (aHR 1.903; 95% CI 1.203–3.010; *p* = 0.006) and any repeat revascularization rate (aHR 3.248; 95% CI 1.539–6.854; *p* = 0.002) were significantly lower in high-intensity users than in low-moderate-intensity users (Table 3). As mentioned, in this study, we compared major clinical outcomes between the before and after October 2012 groups according to the enrolled date of individual patient (Additional file 1: 2, 3, 4 and 5). The trend of change in the major clinical outcomes shown in Additional file 1: 2, 3, 4 and 5 were of similar those shown in Tables 2 and 3. In high-intensity statin users, the values of aHR for MACE and any repeat revascularization in after October 2012 group were low than those in before October 2012 group (e.g. 2.635 vs. 1.845 or 4.162 vs. 2.845, Additional file 1: 2 and 3). Hence, we can assume patients enrolled later may have benefited from innovative therapies that may have impacted prognosis. Although there are some debates [26, 27], lipophilic statins (atorvastatin, simvastatin, pitavastatin, and fluvastatin), especially at high intensity, may lead to unfavorable metabolic effects, including reduction of insulin secretion and exacerbation of insulin resistance [28, 29] and hydrophilic statins (rosuvastatin and pravastatin) could reduce the risk of cardiovascular disease compared with lipophilic statins [30, 31]. In our study, the number of patients who received atorvastatin was higher in normoglycemia group than in the prediabetes and T2DM groups (54.6% vs. 44.7% vs. 50.4%, *p* < 0.001, Table 1). Additionally, atorvastatin (aHR 1.578; 95% CI 1.108–2.392; *p* = 0.021) was independent predictors of MACE (Additional file 1: 11) in high-intensity statin users. However, although the number of patients who received rosuvastatin was lower in normoglycemia group than in the prediabetes and T2DM (38.7% vs. 47.1% vs. 40.3%, *p* < 0.001, Table 1), rosuvastatin was not independent predictor of MACE in this high-intensity statin users (aHR 1.301; 95% CI 1.100–1.775; *p* = 0.101, Additional file 1: 11). Despite the fact that statin treatments can improve endothelial function, increase the bioavailability of nitric oxide, and produce antioxidant and anti-inflammatory effects [32], hyperglycemia accelerates the formation of advanced glycation end products (AGEs) by nonenzymatic glycation reactions [33]. Therefore, hyperglycemia and increased AGE formation lead to tissue damage and cardiovascular complications [34]. Hence, our results suggest that hyperglycemic status may be more related to poor clinical outcomes than with normoglycemia, even after higher-intensity statin treatment. However, this hypothesis is likely to be proved by further studies.

Patients with DM are at intermediate or high risk of atherosclerotic cardiovascular disease [35, 36]. In contrast, in the era of newer-generation DES, the clinical significance of prediabetes in patients with AMI is not well understood. Huang et al. [37] reported that prediabetes defined by HbA1c was associated with an increased risk of composite cardiovascular events (relative risk: 1.21, 95% CI 1.01–1.44), and the health risk increased in patients with an FPG concentration as low as 5.6 mmol/L (100 mg/dL) in their meta-analysis study. Chronically elevated glucose leads to pan-vascular damage, which is present in the prediabetic state, and its severity is determined by the time of hyperglycemia onset [38, 39]. The period between waiting for hyperglycemia to reach the currently accepted cutoff levels for the diagnosis of T2DM and to intervene, may allow vascular damage to advance and become irreversible [40]. Therefore, patients with prediabetes could show worse outcomes compared with those with normoglycemia. Hence, our results showing comparable clinical outcomes between the prediabetes and T2DM groups in both high-intensity and low-moderate-intensity statin users are consistent with recent reports [12, 13]. According to a recent published report [41] after statin treatment, the cumulative incidences of MACE (*p* = 0.314), all-cause death (*p* = 0.530), cardiac death (*p* = 0.873), Re-MI (*p* = 0.170), and any repeat revascularization (*p* = 0.548) were similar between the prediabetes and T2DM groups regardless of statin intensity.

Radial access has proved to be beneficial in reducing the incidence of hemorrhagic events, mortality and acute kidney injury compared to femoral access [42]. In our study, the number of cases with transradial or transfemoral approaches was not significantly different between high-intensity and low-moderate-intensity statin treatment or between statin users and nonusers.

Finally, Gragnano et al. [43] suggested that proprotein convertase subtilisin/kexin 9 inhibitors (PCSK 9i) may represent an attractive strategy to overcome nonadherence barriers in selected high-risk patients. In the recent report [44], PCSK9i improved the quality of life and global health status of patients at high or very high cardiovascular risk, beyond their LDL-cholesterol lowering and positive prognostic impact. Therefore, the use of non-statin drugs may help in increasing adherence to statins.

In this retrospective registry study, more than 50 high-volume university or community hospitals of South Korea were included [14]. As mentioned, in many previous studies, the patients were not confined to AMI [2–4], and they received first-generation DES [2, 9]. Therefore, their results might not reflect contemporary practice using second-generation DES. However, our study population was strictly confined to patients who received newer-generation DES. Moreover, studies concerning the long-term effects of statin therapy in patients with AMI and prediabetes are very limited. Hence, we believe that our study can provide useful information to interventional cardiologists performing PCI with new-generation DES in AMI patients, regarding the importance of hyperglycemia (especially prediabetes) and the relationship with worse cardiovascular outcomes after both high-intensity and low-moderate-intensity statin treatment.

This study has several limitations. First, because of the lack of information in the KAMIR data, we could not present the cumulative events of statin-related new-onset DM during the follow-up period. This is a major weakness of this study. Second, Gragnano et al. [45] mentioned that insufficient LDL-C reduction and high residual risk in a significant proportion of statin-treated patients signify that additional therapies are required to deliver more effective coronary care. Therefore, LDL-cholesterol levels are important during the follow-up period. However, we could not provide these values due to the limitation of this registry data. Third, we did not perform oral glucose tolerance to define prediabetes, which is an important bias. Fourth, there may have been some under-reporting and/or missing data due to the registry nature of this study. Fifth, treatment adherence remains essential in the management of patients with AMI undergoing PCI [45, 46]. Especially, DAPT is recommended for at least 12 months in patients after an acute coronary syndrome (ACS). Underuse or premature discontinuations of DAPT are common in clinical practice. Currently, Crisci et al. [46] are investigating the impact of a dedicated follow-up strategy with clinical visits and counseling on adherence levels to ticagrelor in patients with ACS through a PROGRESS (PROmotinG dual antiplatelet therapy adheREnce in the setting of acute coronary Syndromes) prospective randomized trial. However, because this study was based on discharge medications, we could not precisely determine the adherence or non-adherence of the enrolled patients to their prescribed discharge medications during the follow-up period; this might constitute an additional bias. Moreover, recent antidiabetic medications have been shown to improve cardiovascular outcomes [47–49]. Especially, sodium-glucose co-transporter 2 (SGLT-2) inhibitors, initially introduced for the treatment of DM, demonstrates cardiovascular and renal benefit in patients with heart failure (HF) [47]. Lu et al. [48] demonstrated that beneficial effects SGLT-2 inhibitors were robust in HF patients regardless of T2DM status, and a strong trend to be effective in HF with preserved EF. Recent review [49] also introduced that the hypotheses on SGLT-2 inhibitors mechanisms of action have changed: from simple glycosuric drugs, with consequent glucose lowering, erythropoiesis enhancing and ketogenesis stimulating, to intracellular sodium-lowering molecules. However, unfortunately, this registry data did not include information concerning SGLT-2 inhibitors. Hence, we could not provide comparative cardiovascular effects of SGLT-2 inhibitors between high-intensity and low-moderate-intensity statin treatment or between statin users and nonusers in our study. In addition, diabetic patients may benefit from long-term antiplatelet therapy, and diabetes is a key criterion for choosing continuation of DAPT in life [50]. Cesaro et al. [50] showed that in a real-world study, including patients with previous MI, low-dose ticagrelor for prolonged dual antiplatelet therapy showed to be effective and safe, with no major bleeding occurring at follow-up. Therefore, the duration and kinds of DAPT in patients with AMI is very important. However, because of limitation on registry data, the information requested was not available. This might constitute an important shortcoming of this study. Sixth, although multivariate analysis was performed to strengthen our results, variables not included in the KAMIR may cause a bias. Finally, this study encompasses a very broad time frame (2005 to 2015). Although we stratified patients into two groups before and after October 2012 according to the enrolled date of individual patient, this factor may lead to a bias.

## Conclusions

In this retrospective registry study, prediabetes and T2DM groups showed comparable clinical outcomes, after both high-intensity and low-moderate-intensity statin treatments. Moreover, the beneficial effects of high-intensity compared to low-moderate-intensity statin therapy were more apparent in the normoglycemia group than in the prediabetes and T2DM groups. However, these results are likely to be clearly proved by further studies, especially in patients with AMI who are being treated in contemporary practice.

## Supplementary Information

**Additional file 1**. Supplementary Appendix.

## Data Availability

All data generated or analysed during this study are included in this published article. And any additional data/files may be obtained from the corresponding author on reasonable request.

## Abbreviations

AMI: Acute myocardial infarction
KAMIR: Korea AMI registry
PCI: Percutaneous coronary intervention
T2DM: Type 2 diabetes mellitus
MACE: Major adverse cardiac events
DES: Drug-eluting stent
Re-MI: Recurrent myocardial infarction
ACEIs: Angiotensin-converting enzyme inhibitors
LVEF: Left ventricular ejection fraction
eGFR: Estimated glomerular filtration rate
HbA1c: Hemoglobin A1c
