# Supplementary material for: Comparative effect of statin intensity between prediabetes and type 2 diabetes mellitus after implanting newer-generation drug-eluting stents in Korean acute myocardial infarction patients: a retrospective observational study
Source: BMC Cardiovasc Disord. 2021 Aug 9;21:386. doi: 10.1186/s12872-021-02198-w (PMC8351104; doi:10.1186/s12872-021-02198-w)
Supplement: Supplementary file 1 — Additional file 1. Supplementary Appendix. [file 12872_2021_2198_MOESM1_ESM.docx]

**Supplementary Appendix**

**Comparative effect of statin intensity between prediabetes and type 2 diabetes mellitus after implanting newer-generation drug-eluting stents in Korean acute myocardial infarction patients**

**Running title:** Prediabetic vs. type 2 diabetic AMI based on statin intensity

Yong Hoon Kim^1,*^, Ae-Young Her^1^, Myung Ho Jeong^2^, Byeong-Keuk Kim^3^, Sung-Jin Hong^3^, Seunghwan Kim^4^, Chul-Min Ahn^3^, Jung-Sun Kim^3^, Young-Guk Ko^3^, Donghoon Choi^3^, Myeong-Ki Hong^3^ and Yangsoo Jang^3^

**Supplementary Online Materials**

**Supplementary material 1.** Baseline characteristics in statin non-users

**Supplementary material 2.** Clinical outcomes in high-intensity or low-moderate-intensity statin users before October 2012

**Supplementary material 3.** Clinical outcomes in high-intensity or low-moderate-intensity statin users after October 2012

**Supplementary material 4.** Clinical outcomes between high-intensity and low-moderate-intensity statin in three different glycemic statuses before October 2012

**Supplementary material 5.** Clinical outcomes between high-intensity and low-moderate-intensity statin in three different glycemic statuses before October 2012

**Supplementary material 6.** Univariate analysis for MACE in high-intensity or low-moderate-intensity statin users

**Supplementary material 7.** Baseline characteristics between high-intensity and low-moderate-intensity statin users

**Supplementary material 8.** Comparison of baseline characteristics between statin users and statin non-users

**Supplementary material 9.** Comparison of baseline characteristics between high-intensity and low-moderate-intensity statin treatment or between statin users and non-users.

**Supplementary material 10.** Clinical outcomes between statin users and nonusers at 2 years **Supplementary material 11.** Multivariate Cox-proportional regression analysis for independent predictor of MACE in high-intensity statin users

**Supplementary material 12.** Multivariate Cox-proportional regression analysis for independent predictor of MACE in low-moderate-intensity statin users

**Supplementary material 1** Baseline characteristics in statin non-users

| Variables | Total  (n = 1719) | Group A1  Normoglycemia  (n = 372) | Group B1  Prediabetes  (n = 508) | Group C1  T2DM  (n = 839) | *p* value |
| --- | --- | --- | --- | --- | --- |
| Age (years) | 64.9 ± 12.6 | 63.1 ± 14.3 | 64.8 ± 12.9 | 65.7 ± 11.6 | 0.004 |
| Male, n (%) | 1215 (70.7) | 288 (77.4) | 372 (73.2) | 555 (66.2) | <0.001 |
| LVEF (%) | 49.7 ± 13.1 | 50.8 ± 13.0 | 50.2 ± 13.0 | 48.9 ± 13.0 | 0.039 |
| BMI (kg/m^2^) | 23.9 ± 3.2 | 23.5 ± 3.2 | 23.7 ± 3.3 | 24.1 ± 3.2 | 0.005 |
| SBP (mmHg) | 127.6 ± 29.0 | 125.2 ± 27.8 | 128.8 ± 28.6 | 128.0 ± 29.7 | 0.156 |
| DBP (mmHg) | 77.5 ± 16.6 | 78.0 ± 16.7 | 78.6 ± 16.1 | 76.7 ± 16.8 | 0.118 |
| STEMI, n (%) | 1018 (59.2) | 237 (63.7) | 324 (63.8) | 457 (54.5) | <0.001 |
| Primary PCI, n (%) | 971 (95.4) | 227 (95.8) | 305 (94.1) | 439 (96.1) | 0.426 |
| NSTEMI, n (%) | 701 (40.8) | 135 (36.3) | 184 (36.2) | 382 (45.5) | <0.001 |
| PCI within 24 hours | 562 (80.2) | 115 (85.2) | 148 (80.4) | 299 (78.3) | 0.222 |
| Cardiogenic shock, n (%) | 113 (6.6) | 22 (5.9) | 26 (5.1) | 65 (7.7) | 0.142 |
| Hypertension, n (%) | 905 (52.6) | 171 (46.0) | 226 (44.5) | 508 (60.5) | <0.001 |
| Dyslipidemia, n (%) | 151 (8.9) | 14 (3.8) | 44 (8.7) | 93 (11.1) | <0.001 |
| Previous MI, n (%) | 56 (3.3) | 10 (2.7) | 9 (1.8) | 37 (4.4) | 0.024 |
| Previous PCI, n (%) | 98 (5.7) | 14 (3.8) | 21 (4.1) | 63 (7.5) | 0.007 |
| Previous CABG, n (%) | 6 (0.3) | 0 (0.0) | 0 (0.0) | 6 (0.7) | 0.043 |
| Previous HF, n (%) | 35 (2.0) | 2 (0.5) | 9 (1.8) | 24 (2.9) | 0.027 |
| Previous CVA, n (%) | 120 (7.0) | 28 (7.5) | 25 (4.9) | 67 (8.0) | 0.091 |
| Current smokers, n (%) | 683 (39.7) | 167 (44.9) | 219 (43.1) | 297 (35.4) | 0.001 |
| Peak CK-MB (mg/dL) | 140.6 ± 232.6 | 173.9 ± 359.3 | 164.0 ± 227.9 | 111.7 ± 143.8 | <0.001 |
| Peak troponin-I (ng/mL) | 49.5 ± 109.6 | 50.2 ± 84.2 | 50.1 ± 83.4 | 48.8 ± 131.5 | 0.970 |
| NT-ProBNP (pg/mL) | 3118.4 ± 4931.1 | 2638.4 ± 4028.9 | 2487.0 ± 3339.8 | 3713.6 ± 5394.3 | <0.001 |
| Hs-CRP (mg/dL) | 15.8 ± 52.9 | 10.8 ± 40.8 | 13.7 ± 45.8 | 19.3 ± 61.0 | 0.022 |
| Serum creatinine (mg/L) | 1.24 ± 1.25 | 1.04 ± 0.47 | 1.17 ± 1.31 | 1.37 ± 1.42 | <0.001 |
| eGFR (mL/min/1.73m^2^) | 80.6 ± 42.7 | 84.1 ± 33.8 | 81.4 ± 30.4 | 78.6 ± 51.7 | 0.105 |
| Blood glucose (mg/L) | 193.0 ± 95.9 | 152.0 ± 73.8 | 152.8 ± 53.1 | 235.8 ± 105.8 | <0.001 |
| Hemoglobin A1c (%) | 6.8 ± 2.7 | 5.3 ± 0.5 | 6.0 ± 0.2 | 7.9 ± 3.5 | <0.001 |
| Total cholesterol (mg/dL) | 174.8 ± 45.1 | 172.0 ± 40.6 | 184.4 ± 46.6 | 170.1 ± 45.2 | <0.001 |
| Triglyceride (mg/L) | 130.0 ± 106.2 | 110.1 ± 76.8 | 125.7 ± 85.0 | 141.5 ± 125.7 | <0.001 |
| HDL cholesterol (mg/L) | 42.5 ± 18.4 | 44.3 ± 19.6 | 43.8 ± 17.8 | 40.8 ± 18.1 | 0.002 |
| LDL cholesterol (mg/L) | 109.3 ± 38.8 | 108.2 ± 37.2 | 115.8 ± 37.6 | 105.8 ± 39.8 | <0.001 |
| Discharge medications |  |  |  |  |  |
| Aspirin, n (%) | 1651 (98.0) | 357 (96.0) | 493 (97.0) | 801 (95.5) | 0.354 |
| Clopidogrel, n (%) | 1579 (91.9) | 331 (89.0) | 478 (94.1) | 770 (91.8) | 0.023 |
| Ticagrelor, n (%) | 40 (2.3) | 16 (4.3) | 7 (1.4) | 17 (2.0) | 0.013 |
| Prasugrel, n (%) | 32 (1.9) | 10 (2.7) | 8 (1.6) | 14 (1.7) | 0.408 |
| Cilostazole, n (%) | 276 (16.1) | 56 (15.1) | 89 (17.5) | 131 (15.6) | 0.547 |
| BBs, n (%) | 1074 (62.5) | 232 (62.4) | 308 (60.6) | 534 (63.6) | 0.540 |
| ACEIs, n (%) | 654 (38.0) | 161 (43.3) | 194 (38.2) | 299 (35.6) | 0.041 |
| ARBs, n (%) | 370 (21.5) | 69 (18.5) | 89 (17.5) | 212 (25.3) | 0.001 |
| CCBs, n (%) | 114 (6.6) | 28 (7.5) | 29 (5.7) | 57 (6.8) | 0.544 |
| Diabetes management |  |  |  |  |  |
| Diet, n (%) | 66/839 (7.9) |  |  | 66 (7.9) |  |
| Oral agent, n (%) | 545/839 (65.0) |  |  | 545 (65.0) |  |
| Insulin, n (%) | 50/839 (6.0) |  |  | 50 (6.0) |  |
| Untreated, n (%) | 178/839 (21.1) | - |  | 178 (21.2) |  |
| Infarct-related artery |  |  |  |  |  |
| Left main, n (%) | 36 (2.1) | 7 (1.9) | 9 (1.8) | 20 (2.4) | 0.711 |
| LAD, n (%) | 844 (49.1) | 179 (48.1) | 267 (52.6) | 398 (47.4) | 0.186 |
| LCx, n (%) | 273 (15.9) | 53 (14.2) | 72 (14.2) | 148 (17.6) | 0.150 |
| RCA, n (%) | 566 (32.9) | 133 (35.8) | 160 (31.5) | 273 (32.5) | 0.392 |
| Treated vessel |  |  |  |  |  |
| Left main, n (%) | 56 (3.3) | 12 (3.2) | 15 (3.0) | 29 (3.5) | 0.880 |
| LAD, n (%) | 974 (56.7) | 208 (55.9) | 294 (57.9) | 472 (56.3) | 0.801 |
| LCx, n (%) | 418 (24.3) | 77 (20.7) | 112 (22.0) | 229 (27.3) | 0.017 |
| RCA, n (%) | 658 (38.3) | 149 (40.1) | 149 (35.2) | 330 (39.3) | 0.237 |
| ACC/AHA lesion type |  |  |  |  |  |
| Type B1, n (%) | 282 (16.4) | 63 (16.9) | 84 (16.5) | 135 (16.1) | 0.931 |
| Type B2, n (%) | 482 (28.0) | 88 (23.7) | 153 (30.1) | 241 (28.7) | 0.090 |
| Type C, n (%) | 670 (39.0) | 151 (40.6) | 181 (35.6) | 338 (40.3) | 0.182 |
| Extent of CAD |  |  |  |  |  |
| Single-vessel, n (%) | 799 (46.5) | 194 (52.2) | 251 (49.4) | 354 (42.2) | 0.002 |
| Two-vessel, n (%) | 526 (30.6) | 112 (30.1) | 156 (30.7) | 258 (30.8) | 0.973 |
| ≥ Three-vessel, n (%) | 394 (22.9) | 66 (17.7) | 101 (19.9) | 227 (27.1) | <0.001 |
| Vascular access |  |  |  |  |  |
| Transradial, n (%) | 479 (27.9) | 111 (29.8) | 147 (28.9) | 221 (26.3) | 0.371 |
| Transfemoral, n (%) | 1240 (72.1) | 261 (70.2) | 361 (71.1) | 618 (73.7) | 0.371 |
| IVUS, n (%) | 347 (20.2) | 88 (23.7) | 98 (19.3) | 161 (19.2) | 0.170 |
| OCT, n (%) | 9 (0.5) | 2 (0.5) | 2 (0.4) | 5 (0.6) | 0.882 |
| FFR, n (%) | 3 (0.2) | 1 (0.3) | 0 (0.0) | 2 (0.2) | 0.529 |
| Drug-eluting stents^a^ |  |  |  |  |  |
| ZES, n (%) | 651 (37.9) | 141 (37.9) | 193 (38.0) | 317 (37.8) | 0.997 |
| EES, n (%) | 864 (50.3) | 188 (50.5) | 266 (52.4) | 410 (48.9) | 0.458 |
| BES, n (%) | 175 (10.2) | 37 (9.9) | 41 (8.1) | 97 (11.6) | 0.120 |
| Others, n (%) | 44 (2.6) | 10 (2.7) | 10 (2.0) | 24 (2.9) | 0.594 |
| Stent diameter (mm) | 3.12 ± 0.43 | 3.21 ± 0.42 | 3.15 ± 0.44 | 3.07 ± 0.42 | <0.001 |
| Stent length (mm) | 25.4 ± 9.14 | 25.4 ± 8.99 | 24.9 ± 7.61 | 25.7 ± 10.0 | 0.373 |
| Number of stent | 1.46 ± 0.76 | 1.41 ± 0.74 | 1.42 ± 0.74 | 1.51 ± 0.78 | 0.034 |

Values are means ± SD or numbers and percentages. The *p* values for continuous data obtained from the analysis of variance. The *p* values for categorical data from chi-square or Fisher’s exact test. LVEF: left ventricular ejection fraction; BMI: body mass index; SBP: systolic blood pressure; DBP: diastolic blood pressure; STEMI: ST-elevation myocardial infarction; NSTEMI: non-ST-elevation myocardial infarction; PCI: percutaneous coronary intervention; CABG: coronary artery bypass graft; HF: heart failure; CVA: cerebrovascular accident; CK-MB: creatine kinase myocardial band; NT-ProBNP: N-terminal pro-brain natriuretic peptide; hs-CRP: high sensitivity C-reactive protein; eGFR: estimated glomerular filtration rate; HDL: high-density lipoprotein; LDL: low-density lipoprotein; BBs: beta-blockers; ACEs: angiotensin converting enzyme inhibitors; ARBs: angiotensin receptor blockers; CCBs: calcium channel blockers; IRA: infarct-related artery; LAD: left anterior descending coronary artery; LCx: left circumflex coronary artery; RCA: right coronary artery; ACC/AHA: American College of Cardiology/American Heart Association; CAD: coronary artery disease; IVUS: intravascular ultrasound; OCT: optical coherence tomography; FFR: fractional flow reserve; ZES: zotarolimus-eluting stent; EES: everolimus-eluting stent; BES: biolimus-eluting stents.

^a^Drug-eluting stents were composed of ZES (Resolute Integrity stent; Medtronic, Inc., Minneapolis, MN), EES (Xience Prime stent, Abbott Vascular, Santa Clara, CA; or Promus Element stent, Boston Scientific, Natick, MA), BES (BioMatrix Flex stent, Biosensors International, Morges, Switzerland; or Nobori stent, Terumo Corporation, Tokyo, Japan), and others include any other newer-generation drug-eluting stents except for ZES, EES, and BES.

**Supplementary material 2** Clinical outcomes in high-intensity or low-moderate-intensity statin users before October 2012

| High-intensity statin (n = 1451) | | | | | | | |
| --- | --- | --- | --- | --- | --- | --- | --- |
|  | Group A1  Normoglycemia  (n = 334) | Group A2  Prediabetes  (n = 486) | Log-Rank | Unadjusted | | Adjusted^a^ | |
|  |  |  |  | HR (95% CI) | *p* value | HR (95% CI) | *p* value |
| MACE | 12 (3.6) | 43 (8.8) | 0.003 | 2.516 (1.327-4.772) | 0.005 | 2.635 (1.419-5.128) | 0.003 |
| All-cause death | 5 (1.5) | 16 (3.3) | 0.110 | 2.217 (0.812-6.051) | 0.120 | 2.288 (0.818-6.400) | 0.115 |
| Cardiac death | 3 (0.9) | 12 (2.5) | 0.100 | 2.760 (0.779-9.780) | 0.116 | 3.067 (0.826-11.39) | 0.094 |
| Re-MI | 5 (1.5) | 8 (1.7) | 0.856 | 1.109 (0.363-3.390) | 0.856 | 1.018 (0.320-3.239) | 0.976 |
| Any repeat revascularization | 4 (1.1) | 20 (4.2) | 0.014 | 3.528 (1.206-10.32) | 0.021 | 4.162 (1.395-13.97) | 0.002 |
|  | Group A1  Normoglycemia  (n = 334) | Group A3  T2DM  (n = 631) | Log-Rank | Unadjusted | | Adjusted^a^ | |
|  |  |  |  | HR (95% CI) | *p* value | HR (95% CI) | *p* value |
| MACE | 12 (3.6) | 65 (10.3) | <0.001 | 2.999 (1.565-5.372) | 0.001 | 2.896 (1.557-5.342) | 0.002 |
| All-cause death | 5 (1.5) | 28 (4.4) | 0.017 | 3.002 (1.159-7.714) | 0.024 | 1.992 (0.808-5.107) | 0.109 |
| Cardiac death | 3 (0.9) | 21 (3.3) | 0.022 | 3.737 (1.115-12.53) | 0.033 | 2.374 (0.678-8.313) | 0.176 |
| Re-MI | 5 (1.5) | 13 (2.1) | 0.529 | 1.391 (0.496-3.901) | 0.531 | 1.594 (0.534-4.754) | 0.403 |
| Any repeat revascularization | 4 (1.1) | 27 (4.4) | 0.009 | 3.667 (1.283-10.48) | 0.015 | 4.666 (1.570-14.12) | 0.001 |
|  | Group A2  Prediabetes  (n = 486) | Group A3  T2DM  (n = 631) | Log-Rank | Unadjusted | | Adjusted^a^ | |
|  |  |  |  | HR (95% CI) | *p* value | HR (95% CI) | *p* value |
| MACE | 43 (8.8) | 65 (10.3) | 0.441 | 1.145 (0.778-1.685) | 0.492 | 1.071 (0.703-1.603) | 0.718 |
| All-cause death | 16 (3.3) | 28 (4.4) | 0.340 | 1.347 (0.729-2.489) | 0.342 | 1.022 (0.536-1.910) | 0.803 |
| Cardiac death | 12 (2.5) | 21 (3.3) | 0.409 | 1.346 (0.662-2.736) | 0.411 | 1.020 (0.498-2.140) | 0.753 |
| Re-MI | 8 (1.7) | 13 (2.1) | 0.616 | 1.252 (0.519-3.021) | 0.617 | 1.441 (0.582-3.571) | 0.429 |
| Any repeat revascularization | 20 (4.2) | 27 (4.4) | 0.896 | 1.039 (0.583-1.853) | 0.896 | 1.037 (0.575-1.871) | 0.904 |
| Low-moderate-intensity statin (n = 3910) | | | | | | | |
|  | Group B1  Normoglycemia  (n = 834) | Group B2  Prediabetes  (n = 1311) | Log-Rank | Unadjusted |  | Adjusted^b^ |  |
|  |  |  |  | HR (95% CI) | *p* value | HR (95% CI) | *p* value |
| MACE | 52 (6.2) | 94 (7.2) | 0.398 | 1.157 (0.824-1.623) | 0.399 | 1.155 (0.820-1.626) | 0.409 |
| All-cause death | 19 (2.3) | 37 (2.8) | 0.441 | 1.242 (0.714-2.159) | 0.443 | 1.258 (0.719-2.202) | 0.421 |
| Cardiac death | 15 (1.8) | 29 (2.2) | 0.510 | 1.232 (0.660-2.298) | 0.512 | 1.213 (0.645-2.281) | 0.548 |
| Re-MI | 9 (1.1) | 18 (1.4) | 0.542 | 1.282 (0.576-2.853) | 0.543 | 1.252 (0.558-2.811) | 0.586 |
| Any repeat revascularization | 28 (3.4) | 40 (3.1) | 0.712 | 1.095 (0.676-1.775) | 0.713 | 1.102 (0.676-1.795) | 0.697 |
|  | Group B1  Normoglycemia  (n = 834) | Group B3  T2DM  (n = 1765) | Log-Rank | Unadjusted |  | Adjusted^b^ |  |
|  |  |  |  | HR (95% CI) | *p* value | HR (95% CI) | *p* value |
| MACE | 52 (6.2) | 155 (8.8) | 0.027 | 1.422 (1.039-1.947) | 0.028 | 1.372 (1.011-1.851) | 0.053 |
| All-cause death | 19 (2.3) | 61 (4.5) | 0.106 | 1.524 (0.911-2.550) | 0.109 | 1.621 (1.102-2.614) | 0.044 |
| Cardiac death | 15 (1.8) | 38 (2.2) | 0.546 | 1.201 (0.661-2.183) | 0.548 | 1.156 (0.598-2.065) | 0.682 |
| Re-MI | 9 (1.1) | 37 (2.1) | 0.064 | 1.964 (0.948-4.070) | 0.069 | 1.875 (0.910-3.593) | 0.072 |
| Any repeat revascularization | 28 (3.4) | 64 (3.7) | 0.705 | 1.089 (0.699-1.699) | 0.705 | 1.073 (0.679-1.687) | 0.725 |
|  | Group B2  Prediabetes  (n = 1311) | Group B3  T2DM  (n = 1765) | Log-Rank | Unadjusted |  | Adjusted^b^ | |
|  |  |  |  | HR (95% CI) | *p* value | HR (95% CI) | *p* value |
| MACE | 94 (7.2) | 155 (8.8) | 0.133 | 1.229 (0.951-1.588) | 0.114 | 1.198 (0.902-1.524) | 0.201 |
| All-cause death | 37 (2.8) | 61 (4.5) | 0.326 | 1.226 (0.815-1.845) | 0.328 | 1.422 (1.042-2.047) | 0.054 |
| Cardiac death | 29 (2.2) | 38 (2.2) | 0.915 | 1.027 (0.633-1.664) | 0.915 | 1.110 (0.674-1.830) | 0.761 |
| Re-MI | 18 (1.4) | 37 (2.1) | 0.135 | 1.531 (0.872-2.689) | 0.138 | 1.497 (0.798-2.583) | 0.164 |
| Any repeat revascularization | 40 (3.1) | 64 (3.7) | 0.379 | 1.194 (0.804-1.772) | 0.380 | 1.157 (0.777-1.715) | 0.604 |

^a^ Adjusted by age, male, LVEF, cardiogenic shock, STEMI, hypertension, previous MI, previous CVA, current smoker, NT-ProBNP, serum creatinine, eGFR, atorvastatin, ACC/AHA type B2 lesion, ≥ Three-vessel disease, and number of stent

^b^ Adjusted by age, male, LVEF, cardiogenic shock, STEMI, hypertension, previous MI, previous CVA, current smoker, NT-ProBNP, serum creatinine, eGFR, total cholesterol, beta-blocker, ACEI, ARB, rosuvastatin, simvastatin, intravascular ultrasound, single-vessel disease, ≥ three-vessel disease, and number of stent.

HR: hazard ratio; CI: confidence interval; T2DM: type 2 diabetes mellitus; MACE: major adverse cardiac events; Re-MI: recurrent myocardial infarction; LVEF: left ventricular ejection fraction; STEMI: ST-segment elevation myocardial infarction; CVA: cerebrovascular accidents; NT-ProBNP: N-terminal pro-brain natriuretic peptide; eGFR: estimated glomerular filtration rate; ACC/AHA: American College of Cardiology/American Heart Association; ACEI: angiotensin-converting enzyme inhibitors; ARB: angiotensin receptor blockers.

**Supplementary material 3** Clinical outcomes in high-intensity or low-moderate-intensity statin users after October 2012

| High-intensity statin (n = 1533) | | | | | | | |
| --- | --- | --- | --- | --- | --- | --- | --- |
|  | Group A1  Normoglycemia  (n = 472) | Group A2  Prediabetes  (n = 449) | Log-Rank | Unadjusted | | Adjusted^a^ | |
|  |  |  |  | HR (95% CI) | *p* value | HR (95% CI) | *p* value |
| MACE | 11 (3.3) | 18 (4.9) | 0.145 | 1.734 (0.819-3.671) | 0.150 | 1.845 (0.984-3.997) | 0.048 |
| All-cause death | 3 (0.7) | 5 (1.5) | 0.433 | 1.760 (0.421-7.365) | 0.439 | 1.194 (0.260-5.484) | 0.820 |
| Cardiac death | 2 (0.4) | 3 (0.9) | 0.606 | 1.594 (0.266-9.538) | 0.610 | 1.038 (0.134-8.025) | 0.871 |
| Re-MI | 4 (1.6) | 8 (2.4) | 0.224 | 2.073 (0.624-6.886) | 0.234 | 2.280 (0.638-8.146) | 0.205 |
| Any repeat revascularization | 4 (1.1) | 8 (2.2) | 0.207 | 2.128 (0.641-7.067) | 0.218 | 2.845 (0.984-9.841) | 0.044 |
|  | Group A1  Normoglycemia  (n = 472) | Group A3  T2DM  (n = 612) | Log-Rank | Unadjusted | | Adjusted^a^ | |
|  |  |  |  | HR (95% CI) | *p* value | HR (95% CI) | *p* value |
| MACE | 11 (3.3) | 31 (7.5) | 0.019 | 2.216 (1.117-4.395) | 0.023 | 2.146 (1.063-4.335) | 0.033 |
| All-cause death | 3 (0.7) | 13 (2.9) | 0.049 | 3.287 (0.937-11.54) | 0.063 | 2.713 (0.742-9.921) | 0.131 |
| Cardiac death | 2 (0.4) | 7 (1.7) | 0.207 | 2.648 (0.550-12.75) | 0.225 | 1.844 (0.349-9.746) | 0.471 |
| Re-MI | 4 (1.6) | 16 (6.8) | 0.039 | 2.999 (1.003-8.972) | 0.049 | 2.813 (0.922-8.585) | 0.069 |
| Any repeat revascularization | 4 (1.1) | 15 (3.1) | 0.051 | 2.858 (0.949-8.612) | 0.062 | 3.241 (1.055-9.961) | 0.040 |
|  | Group A2  Prediabetes  (n = 449) | Group A3  T2DM  (n = 612) | Log-Rank | Unadjusted | | Adjusted^a^ | |
|  |  |  |  | HR (95% CI) | *p* value | HR (95% CI) | *p* value |
| MACE | 18 (4.9) | 31 (7.5) | 0.397 | 1.283 (0.720-2.285) | 0.398 | 1.178 (0.649-1.985) | 0.684 |
| All-cause death | 5 (1.5) | 13 (2.9) | 0.220 | 1.887 (0.673-5.294) | 0.228 | 1.580 (0.530-4.709) | 0.412 |
| Cardiac death | 3 (0.9) | 7 (1.7) | 0.440 | 1.694 (0.438-6.553) | 0.445 | 1.193 (0.258-5.522) | 0.821 |
| Re-MI | 8 (2.4) | 16 (6.8) | 0.334 | 1.515 (0.648-3.542) | 0.338 | 1.340 (0.565-3.177) | 0.506 |
| Any repeat revascularization | 8 (2.4) | 15 (3.1) | 0.494 | 1.348 (0.571-3.179) | 0.495 | 1.104 (0.417-2.432) | 0.719 |
| Low-moderate-intensity statin (n = 2999) | | | | | | | |
|  | Group B1  Normoglycemia  (n = 981) | Group B2  Prediabetes  (n = 834) | Log-Rank | Unadjusted |  | Adjusted^b^ |  |
|  |  |  |  | HR (95% CI) | *p* value | HR (95% CI) | *p* value |
| MACE | 52 (6.7) | 45 (6.2) | 0.917 | 1.022 (0.685-1.523) | 0.917 | 1.048 (0.701-1.568) | 0.818 |
| All-cause death | 9 (1.0) | 11 (1.6) | 0.463 | 1.389 (0.576-3.353) | 0.465 | 1.379 (0.566-3.358) | 0.479 |
| Cardiac death | 5 (0.6) | 5 (0.7) | 0.839 | 1.137 (0.329-3.927) | 0.839 | 1.087 (0.310-3.817) | 0.896 |
| Re-MI | 18 (2.7) | 17 (2.3) | 0.829 | 1.076 (0.554-2.088) | 0.829 | 1.031 (0.528-2.013) | 0.929 |
| Any repeat revascularization | 32 (4.4) | 20 (2.9) | 0.198 | 1.440 (0.824-2.518) | 0.201 | 1.219 (0.846-1.755) | 0.288 |
|  | Group B1  Normoglycemia  (n = 981) | Group B3  T2DM  (n = 1184) | Log-Rank | Unadjusted |  | Adjusted^b^ |  |
|  |  |  |  | HR (95% CI) | *p* value | HR (95% CI) | *p* value |
| MACE | 52 (6.7) | 99 (10.0) | 0.009 | 1.560 (1.115-2.183) | 0.009 | 1.429 (1.001-1.998) | 0.043 |
| All-cause death | 9 (1.0) | 38 (3.7) | <0.001 | 3.461 (1.674-7.158) | 0.001 | 2.940 (1.388-6.225) | 0.005 |
| Cardiac death | 5 (0.6) | 25 (2.5) | 0.002 | 4.100 (1.570-10.71) | 0.004 | 3.319 (1.235-8.919) | 0.017 |
| Re-MI | 18 (2.7) | 32 (3.5) | 0.213 | 1.441 (0.809-2.567) | 0.215 | 1.444 (0.785-2.654) | 0.237 |
| Any repeat revascularization | 32 (4.4) | 38 (3.9) | 0.890 | 1.034 (0.646-1.654) | 0.890 | 1.140 (0.698-1.862) | 0.601 |
|  | Group B2  Prediabetes  (n = 834) | Group B3  T2DM  (n = 1184) | Log-Rank | Unadjusted |  | Adjusted^b^ | |
|  |  |  |  | HR (95% CI) | *p* value | HR (95% CI) | *p* value |
| MACE | 45 (6.2) | 99 (10.0) | 0.007 | 1.592 (1.119-2.264) | 0.010 | 1.403 (0.986-2.004) | 0.061 |
| All-cause death | 11 (1.6) | 38 (3.7) | 0.006 | 2.483 (1.269-4.858) | 0.008 | 1.945 (0.981-3.889) | 0.057 |
| Cardiac death | 5 (0.7) | 25 (2.5) | 0.005 | 3.577 (1.369-9.344) | 0.009 | 2.757 (1.038-7.327) | 0.042 |
| Re-MI | 17 (2.3) | 32 (3.5) | 0.316 | 1.350 (0.749-2.430) | 0.318 | 1.332 (0.726-2.423) | 0.354 |
| Any repeat revascularization | 20 (2.9) | 38 (3.9) | 0.236 | 1.385 (0.806-2.381) | 0.238 | 1.266 (0.725-2.211) | 0.407 |

^a^ Adjusted by age, male, LVEF, cardiogenic shock, STEMI, hypertension, previous MI, previous CVA, current smoker, NT-ProBNP, serum creatinine, eGFR, atorvastatin, ACC/AHA type B2 lesion, ≥ Three-vessel disease, and number of stent

^b^ Adjusted by age, male, LVEF, cardiogenic shock, STEMI, hypertension, previous MI, previous CVA, current smoker, NT-ProBNP, serum creatinine, eGFR, total cholesterol, beta-blocker, ACEI, ARB, rosuvastatin, simvastatin, intravascular ultrasound, single-vessel disease, ≥ three-vessel disease, and number of stent.

HR: hazard ratio; CI: confidence interval; T2DM: type 2 diabetes mellitus; MACE: major adverse cardiac events; Re-MI: recurrent myocardial infarction; LVEF: left ventricular ejection fraction; STEMI: ST-segment elevation myocardial infarction; CVA: cerebrovascular accidents; NT-ProBNP: N-terminal pro-brain natriuretic peptide; eGFR: estimated glomerular filtration rate; ACC/AHA: American College of Cardiology/American Heart Association; ACEI: angiotensin-converting enzyme inhibitors; ARB: angiotensin receptor blockers.

**Supplementary material 4** Clinical outcomes between high-intensity and low-moderate-intensity statin in three different glycemic statuses before October 2012

| Outcomes | High-intensity  (n = 334) | Low-moderate-intensity (n = 834) | Log-rank | Unadjusted  HR (95% CI) |  | Adjusted^a^  HR (95% CI) |  |
| --- | --- | --- | --- | --- | --- | --- | --- |
|  |  |  |  |  | *p* value |  | *p* value |
| Normoglycemia | Group A1 | Group B1 |  |  |  |  |  |
| MACE | 12 (3.6) | 52 (6.2) | 0.075 | 1.753 (0.936-3.284) | 0.080 | 1.764 (0.936-3.326) | 0.079 |
| All-cause death | 5 (1.5) | 19 (2.3) | 0.395 | 1.527 (0.570-4.089) | 0.400 | 1.465 (0.542-3.961) | 0.452 |
| Cardiac death | 3 (0.9) | 15 (1.8) | 0.260 | 2.007 (0.581-6.932) | 0.271 | 1.969 (0.561-6.911) | 0.290 |
| Re-MI | 5 (1.5) | 9 (1.1) | 0.557 | 1.385 (0.464-4.133) | 0.559 | 1.353 (0.444-4.119) | 0.595 |
| Any revascularization | 4 (1.1) | 28 (3.4) | 0.041 | 2.845 (0.998-8.111) | 0.050 | 3.025 (1.045-8.760) | 0.041 |
| Outcomes | High-intensity  (n = 486) | Low-moderate-intensity (n = 1311) | Log-rank | Unadjusted  HR (95% CI) |  | Adjusted^a^  HR (95% CI) |  |
|  |  |  |  |  | *p* value |  | *p* value |
| Prediabetes | Group A2 | Group B2 |  |  |  |  |  |
| MACE | 43 (8.8) | 94 (7.2) | 0.238 | 1.242 (0.866-1.782) | 0.239 | 1.232 (0.854-1.776) | 0.265 |
| All-cause death | 16 (3.3) | 37 (2.8) | 0.603 | 1.168 (0.650-2.099) | 0.605 | 1.221 (0.675-2.210) | 0.509 |
| Cardiac death | 12 (2.5) | 29 (2.2) | 0.748 | 1.116 (0.570-2.187) | 0.749 | 1.102 (0.558-2.177) | 0.779 |
| Re-MI | 8 (1.7) | 18 (1.4) | 0.670 | 1.198 (0.521-2.756) | 0.670 | 1.222 (0.482-2.611) | 0.789 |
| Any revascularization | 20 (4.2) | 40 (3.1) | 0.261 | 1.359 (0.794-2.324) | 0.263 | 1.329 (0.984-1.913) | 0.962 |
| Outcomes | High-intensity  (n = 631) | Low-moderate-intensity (n = 1765) | Log-rank | Unadjusted  HR (95% CI) |  | Adjusted^a^  HR (95% CI) |  |
|  |  |  |  |  | *p* value |  | *p* value |
| T2DM | Group A3 | Group B3 |  |  |  |  |  |
| MACE | 65 (10.3) | 155 (8.8) | 0.320 | 1.159 (0.866-1.551) | 0.320 | 1.152 (0.854-1.548) | 0.325 |
| All-cause death | 28 (4.4) | 61 (4.5) | 0.271 | 1.284 (0.821-2.009) | 0.273 | 1.357 (0.862-2.135) | 0.187 |
| Cardiac death | 21 (3.3) | 38 (2.2) | 0.106 | 1.545 (0.907-2.633) | 0.109 | 1.663 (0.970-2.852) | 0.065 |
| Re-MI | 13 (2.1) | 37 (2.1) | 0.963 | 1.015 (0.540-1.909) | 0.963 | 1.025 (0.541-1.940) | 0.941 |
| Any revascularization | 27 (4.4) | 64 (3.7) | 0.466 | 1.182 (0.754-1.853) | 0.466 | 1.147 (0.728-1.808) | 0.554 |
| Outcomes | High-intensity  (n = 1451) | Low-moderate-intensity (n = 3910) | Log-rank | Unadjusted  HR (95% CI) |  | Adjusted^a^  HR (95% CI) |  |
|  |  |  |  |  | *p* value |  | *p* value |
| Total | Group A1+A2+A3 | Group  B1+B2+B3 |  |  |  |  |  |
| MACE | 120 (8.2) | 301 (7.6) | 0.551 | 1.067 (0.883-1.319) | 0.551 | 1.051 (0.849-1.302) | 0.647 |
| All-cause death | 49 (3.4) | 117 (3.0) | 0.478 | 1.128 (0.808-1.575) | 0.479 | 1.130 (0.810-1.582) | 0.474 |
| Cardiac death | 36 (2.5) | 82 (2.1) | 0.400 | 1.182 (0.799-1.750) | 0.402 | 1.156 (0.778-1.715) | 0.473 |
| Re-MI | 26 (1.8) | 64 (1.7) | 0.693 | 1.096 (0.695-1.729) | 0.693 | 1.075 (0.679-1.699) | 0.758 |
| Any revascularization | 51 (3.6) | 132 (3.4) | 0.808 | 1.041 (0.753-1.438) | 0.808 | 1.006 (0.727-1.393) | 0.969 |

^a^Adjusted by age, male, LVEF, BMI, SBP, DBP, cardiogenic shock, hypertension, current smoker, NT-ProBNP, total cholesterol, triglyceride, LDL-cholesterol, ticagrelor, ACEI, ARB, atorvastatin, rosuvastatin, simvastatin, IVUS, and stent diameter

HR: hazard ratio; CI: confidence interval; T2DM: type 2 diabetes mellitus; MACE: major adverse cardiac events; Re-MI: recurrent myocardial infarction; LVEF: left ventricular ejection fraction; BMI: body mass index; SBP: systolic blood pressure; DBP: diastolic blood pressure; NT-ProBNP: N-terminal pro-brain natriuretic peptide; LDL: low-density lipoprotein; ACEI: angiotensin-converting enzyme inhibitors; ARBs: angiotensin receptor blockers; IVUS: intravascular ultrasound.

**Supplementary material 5** Clinical outcomes between high-intensity and low-moderate-intensity statin in three different glycemic statuses after October 2012

| Outcomes | High-intensity  (n = 472) | Low-moderate-intensity (n = 981) | Log-rank | Unadjusted  HR (95% CI) |  | Adjusted^a^  HR (95% CI) |  |
| --- | --- | --- | --- | --- | --- | --- | --- |
|  |  |  |  |  | *p* value |  | *p* value |
| Normoglycemia | Group A1 | Group B1 |  |  |  |  |  |
| MACE | 11 (3.3) | 52 (6.7) | 0.024 | 2.085 (1.088-3.997) | 0.027 | 2.002 (1.025-3.911) | 0.042 |
| All-cause death | 3 (0.7) | 9 (1.0) | 0.621 | 1.388 (0.376-5.130) | 0.623 | 1.213 (0.306-4.812) | 0.784 |
| Cardiac death | 2 (0.4) | 5 (0.6) | 0.847 | 1.174 (0.228-6.054) | 0.848 | 1.476 (0.252-8.629) | 0.666 |
| Re-MI | 4 (1.6) | 18 (2.7) | 0.242 | 1.889 (0.639-5.588) | 0.250 | 2.034 (0.659-6.279) | 0.217 |
| Any revascularization | 4 (1.1) | 32 (4.4) | 0.014 | 3.416 (1.208-9.663) | 0.021 | 3.308 (1.140-9.598) | 0.028 |
| Outcomes | High-intensity  (n = 449) | Low-moderate-intensity (n = 834) | Log-rank | Unadjusted  HR (95% CI) |  | Adjusted^a^  HR (95% CI) |  |
|  |  |  |  |  | *p* value |  | *p* value |
| Prediabetes | Group A2 | Group B2 |  |  |  |  |  |
| MACE | 18 (4.9) | 45 (6.2) | 0.509 | 1.202 (0.696-2.078) | 0.509 | 1.058 (0.602-1.858) | 0.844 |
| All-cause death | 5 (1.5) | 11 (1.6) | 0.937 | 1.044 (0.362-3.008) | 0.937 | 1.066 (0.347-3.237) | 0.911 |
| Cardiac death | 3 (0.9) | 5 (0.7) | 0.736 | 1.279 (0.305-5.361) | 0.736 | 1.130 (0.232-5.513) | 0.880 |
| Re-MI | 8 (2.4) | 17 (2.3) | 0.888 | 1.062 (0.458-2.463) | 0.889 | 1.164 (0.489-2.767) | 0.732 |
| Any revascularization | 8 (2.2) | 20 (2.9) | 0.700 | 1.175 (0.517-2.670) | 0.700 | 1.134 (0.487-2.640) | 0.770 |
| Outcomes | High-intensity  (n = 612) | Low-moderate-intensity (n = 1184) | Log-rank | Unadjusted  HR (95% CI) |  | Adjusted^a^  HR (95% CI) |  |
|  |  |  |  |  | *p* value |  | *p* value |
| T2DM | Group A3 | Group B3 |  |  |  |  |  |
| MACE | 31 (7.5) | 99 (10.0) | 0.052 | 1.481 (0.994-2.207) | 0.054 | 1.325 (0.881-1.994) | 0.176 |
| All-cause death | 13 (2.9) | 38 (3.7) | 0.281 | 1.412 (0.752-2.653) | 0.283 | 1.215 (0.635-2.233) | 0.556 |
| Cardiac death | 7 (1.7) | 25 (2.5) | 0.192 | 1.736 (0.750-4.017) | 0.198 | 1.469 (0.621-3.475) | 0.381 |
| Re-MI | 16 (6.8) | 32 (3.5) | 0.677 | 1.136 (0.622-2.074) | 0.677 | 1.233 (0.665-2.288) | 0.506 |
| Any revascularization | 15 (3.1) | 38 (3.9) | 0.535 | 1.208 (0.664-2.197) | 0.536 | 1.061 (0.574-1.962) | 0.850 |
| Outcomes | High-intensity  (n = 1533) | Low-moderate-intensity (n = 2999) | Log-rank | Unadjusted  HR (95% CI) |  | Adjusted^a^  HR (95% CI) |  |
|  |  |  |  |  | *p* value |  | *p* value |
| Total | Group A1+A2+A3 | Group  B1+B2+B3 |  |  |  |  |  |
| MACE | 60 (5.5) | 196 (7.9) | 0.005 | 1.501 (1.126-2.001) | 0.006 | 1.533 (1.144-2.053) | 0.004 |
| All-cause death | 21 (1.8) | 58 (2.3) | 0.289 | 1.309 (0.794-2.157) | 0.291 | 1.178 (0.707-1.963) | 0.531 |
| Cardiac death | 12 (1.1) | 35 (1.4) | 0.318 | 1.395 (0.724-2.688) | 0.320 | 1.271 (0.650-2.485) | 0.484 |
| Re-MI | 28 (3.9) | 67 (2.9) | 0.753 | 1.074 (0.690-1.670) | 0.753 | 1.082 (0.689-1.699) | 0.731 |
| Any revascularization | 27 (2.2) | 90 (3.8) | 0.051 | 1.529 (0.994-2.351) | 0.053 | 1.587 (1.026-2.456) | 0.038 |

^a^Adjusted by age, male, LVEF, BMI, SBP, DBP, cardiogenic shock, hypertension, current smoker, NT-ProBNP, total cholesterol, triglyceride, LDL-cholesterol, ticagrelor, ACEI, ARB, atorvastatin, rosuvastatin, simvastatin, IVUS, and stent diameter

HR: hazard ratio; CI: confidence interval; T2DM: type 2 diabetes mellitus; MACE: major adverse cardiac events; Re-MI: recurrent myocardial infarction; LVEF: left ventricular ejection fraction; BMI: body mass index; SBP: systolic blood pressure; DBP: diastolic blood pressure; NT-ProBNP: N-terminal pro-brain natriuretic peptide; LDL: low-density lipoprotein; ACEI: angiotensin-converting enzyme inhibitors; ARBs: angiotensin receptor blockers; IVUS: intravascular ultrasound.

**Supplementary material 6** Univariate analysis for MACE in high-intensity or low-moderate-intensity statin users

|  | **High-intensity statin** | | **Low-moderate-intensity statin** | | **Total population** | |
| --- | --- | --- | --- | --- | --- | --- |
| **Variables** | **HR (95% CI)** | ***p* value** | **HR (95% CI)** | ***p* value** | **HR (95% CI)** | ***p* value** |
| Age | 1.017 (1.004 – 1.029) | 0.008 | 1.015 (1.007 – 1.022) | <0.001 | 1.020 (1.015 – 1.025) | <0.001 |
| Male | 1.171 (0.831 – 1.649) | 0.367 | 1.491 (1.238 – 1.794) | <0.001 | 1.438 (1.256 – 1.646) | <0.001 |
| Left ventricular ejection fraction | 0.978 (0.965 – 0.990) | <0.001 | 0.978 (0.971 – 0.986) | <0.001 | 0.968 (0.963 – 0.974) | <0.001 |
| Body mass index | 0.974 (0.929 – 1.022) | 0.285 | 0.976 (0.948 – 1.004) | 0.095 | 0.964 (0.944 – 0.984) | 0.001 |
| Systolic blood pressure | 0.999 (0.994 – 1.005) | 0.839 | 0.997 (0.993 – 1.000) | 0.045 | 0.995 (0.992 – 0.997) | <0.001 |
| Diastolic blood pressure | 0.997 (0.988 – 1.006) | 0.498 | 0.995 (0.990 – 1.001) | 0.104 | 0.993 (0.989 – 0.997) | 0.001 |
| ST-segment elevation MI | 1.331 (0.993 – 1.782) | 0.055 | 1.249 (1.048 – 1.489) | 0.013 | 1.199 (1.056 – 1.361) | 0.005 |
| Cardiogenic shock | 1.355 (0.667 – 2.752) | 0.401 | 1.655 (1.162 – 2.356) | 0.005 | 1.915 (1.510 – 2.427) | <0.001 |
| Primary PCI | 1.156 (0.366 – 3.655) | 0.804 | 1.426 (0.705 – 2.884) | 0.323 | 1.255 (0.689 – 2.289) | 0.458 |
| PCI within 24 hours | 1.654 (0.764 – 3.578) | 0.201 | 1.039 (0.729 – 1.481) | 0.831 | 1.073 (0.779 – 1.479) | 0.665 |
| Hypertension | 1.390 (1.036 – 1.865) | 0.028 | 1.316 (1.102 – 1.572) | 0.002 | 1.275 (1.122 – 1.448) | <0.001 |
| Dyslipidemia | 1.038 (0.652 – 1.652) | 0.876 | 1.225 (0.958 – 1.566) | 0.106 | 1.089 (0.900 – 1.317) | 0.380 |
| Previous MI | 2.673 (1.549 – 4.614) | <0.001 | 1.389 (0.929 – 2.077) | 0.110 | 1.743 (1.331 – 2.283) | <0.001 |
| Previous PCI | 1.331 (0.741 – 2.391) | 0.338 | 1.207 (0.856 – 1.702) | 0.283 | 1.273 (0.995 – 1.629) | 0.055 |
| Previous CABG | 2.955 (0.733 – 11.91) | 0.128 | 2.260 (0.936 – 5.454) | 0.070 | 2.025 (1.010 – 4.062) | 0.047 |
| Previous cerebrovascular accident | 2.203 (1.397 – 3.472) | 0.001 | 1.892 (1.429 – 2.503) | <0.001 | 2.074 (1.704 – 2.524) | <0.001 |
| Previous heart failure | 1.794 (0.445 – 7.228) | 0.411 | 2.581 (0.644 – 10.35) | 0.181 | 1.937 (1.230 – 3.051) | 0.004 |
| Current smokers | 1.417 (1.050 – 1.913) | 0.023 | 1.190 (0.993 – 1.425) | 0.059 | 1.370 (1.201 – 1.563) | <0.001 |
| Peak CK-MB | 0.999 (0.998 – 1.000) | 0.135 | 1.000 (1.000 – 1.001) | 0.634 | 1.000 (0.999 – 1.000) | 0.955 |
| NT-ProBNP | 1.001 (1.000 – 1.002) | <0.001 | 1.001 (1.001 – 1.002) | <0.001 | 1.001 (1.001 – 1.002) | <0.001 |
| Hs-CRP | 1.000 (0.991 – 1.009) | 0.947 | 0.996 (0.992 – 1.000) | 0.067 | 0.999 (0.998 – 1.001) | 0.513 |
| Serum creatinine | 1.107 (1.039 – 1.178) | 0.002 | 1.030 (1.005 – 1.054) | 0.016 | 1.040 (1.025 – 1.055) | <0.001 |
| eGFR | 0.993 (0.989 – 0.998) | 0.007 | 0.994 (0.991 – 0.997) | <0.001 | 0.992 (0.990 – 0.994) | <0.001 |
| Total cholesterol | 0.999 (0.996 – 1.002) | 0.529 | 0.996 (0.994 – 0.998) | <0.001 | 0.995 (0.993 – 0.996) | <0.001 |
| Triglyceride | 1.000 (0.998 – 1.001) | 0.542 | 0.999 (0.997 – 1.000) | 0.005 | 0.999 (0.998 – 1.000) | 0.001 |
| HDL-cholesterol | 0.982 (0.968 – 0.996) | 0.012 | 0.999 (0.993 – 1.006) | 0.792 | 0.988 (0.983 – 0.994) | <0.001 |
| LDL-cholesterol | 1.000 (0.996 – 1.003) | 0.880 | 0.997 (0.994 – 0.999) | 0.015 | 0.995 (0.993 – 0.997) | <0.001 |
| Clopidogrel | 2.338 (1.352 – 4.041) | 0.002 | 1.100 (0.841 – 1.439) | 0.487 | 1.052 (0.870 – 1.272) | 0.600 |
| Ticagrelor | 4.015 (1.647 – 9.786) | 0.002 | 1.001 (0.712 – 1.407) | 0.995 | 1.148 (1.072 – 1.877) | 0.015 |
| Prasugrel | 1.400 (0.657 – 2.981) | 0.383 | 1.393 (0.881 – 2.204) | 0.156 | 1.513 (1.058 – 2.164) | 0.023 |
| Cilostazole | 1.023 (0.707 – 1.480) | 0.906 | 1.157 (0.918 – 1.458) | 0.218 | 1.228 (1.034 – 1.460) | 0.019 |
| Beta-blocker | 1.026 (0.662 – 1.589) | 0.909 | 1.481 (1.180 – 1.859) | 0.001 | 2.380 (2.076 – 2.728) | <0.001 |
| ACEI | 1.423 (1.059 – 1.912) | 0.019 | 1.452 (1.217 – 1.731) | <0.001 | 1.712 (1.506 – 1.946) | <0.001 |
| Angiotensin receptor blocker | 1.371 (1.014 – 1.854) | 0.040 | 1.412 (1.169 – 1.706) | <0.001 | 1.005 (0.870 – 1.161) | 0.946 |
| Calcium channel blocker | 1.333 (0.742 – 2.394) | 0.336 | 1.283 (0.936 – 1.761) | 0.122 | 1.033 (0.800 – 1.334) | 0.801 |
| Atorvastatin | 1.612 (1.203 – 2.428) | 0.009 | 1.105 (0.824 – 1.398) | 0.305 | 1.203 (1.055 – 1.371) | 0.006 |
| Rosuvastatin | 1.232 (0.909 – 1.670) | 0.178 | 1.215 (1.005 – 1.469) | 0.044 | 1.569 (1.350 – 1.824) | <0.001 |
| Simvastatin | 2.308 (0.737 – 7.225) | 0.151 | 1.819 (1.186 – 2.789) | 0.006 | 1.922 (1.292 – 2.859) | 0.001 |
| Right coronary artery (treated) | 1.072 (0.797 – 1.440) | 0.647 | 1.237 (1.036 – 1.476) | 0.019 | 1.085 (0.955 – 1.234) | 0.210 |
| ACC/AHA type B1 lesion | 1.811 (1.030 – 3.184) | 0.039 | 1.210 (0.916 – 1.597) | 0.180 | 1.324 (1.078 – 1.626) | 0.007 |
| ACC/AHA type B2 lesion | 2.010 (1.501 – 2.693) | <0.001 | 1.124 (0.936 – 1.350) | 0.211 | 1.237 (1.085 – 1.410) | 0.001 |
| ACC/AHA type C lesion | 1.397 (1.033 – 1.891) | 0.030 | 1.137 (0.951 – 1.358) | 0.159 | 1.086 (0.956 – 1.234) | 0.205 |
| Intravascular ultrasound | 1.056 (0.750 – 1.488) | 0.755 | 1.437 (1.180 – 1.751) | <0.001 | 1.135 (0.979 – 1.317) | 0.093 |
| Fractional flow reserve | 2.404 (0.337 – 17.16) | 0.382 | 1.120 (0.501 – 2.506) | 0.782 | 1.411 (0.703 – 2.829) | 0.333 |
| Single-vessel disease | 1.369 (1.019 – 1.839) | 0.037 | 1.869 (1.550 – 2.253) | <0.001 | 1.579 (1.385 – 1.800) | <0.001 |
| ≥ Three-vessel disease | 1.569 (1.122 – 2.193) | 0.008 | 2.056 (1.706 – 2.477) | <0.001 | 1.776 (1.538 – 2.027) | <0.001 |
| Zotarolimus-eluting stent | 1.064 (0.782 – 1.447) | 0.694 | 1.017 (0.844 – 1.225) | 0.861 | 1.005 (0.880 – 1.148) | 0.936 |
| Biolimus-eluting stent | 1.159 (0.748 – 1.795) | 0.509 | 1.113 (0.873 – 1.420) | 0.386 | 1.014 (0.844 – 1.218) | 0.882 |
| Stent diameter | 0.880 (0.623 – 1.245) | 0.471 | 0.775 (0.623 – 0.963) | 0.022 | 0.744 (0.641 – 0.874) | <0.001 |
| Stent length | 1.010 (0.999 – 1.021) | 0.076 | 1.003 (0.996 – 1.011) | 0.406 | 1.006 (1.001 – 1.011) | 0.027 |
| Number of stent | 1.298 (1.130 – 1.490) | <0.001 | 1.122 (1.012 – 1.245) | 0.029 | 1.178 (1.098 – 1.263) | <0.001 |

MACE: major adverse cardiac events; HR: hazard ratio; CI: confidence interval; MI: myocardial infarction; PCI: percutaneous coronary intervention; CABG: coronary artery bypass graft; CK-MB: creatine kinase myocardial band; NT-ProBNP: N-terminal pro-brain natriuretic peptide; eGFR: estimated glomerular filtration rate; HDL: high-density lipoprotein; LDL: low-density lipoprotein; ACE: angiotensin converting enzyme inhibitors; ACC/AHA: American College of Cardiology/American Heart Association.

**Supplementary material 7** Baseline characteristics between high-intensity and low-moderate-intensity statin users

| Variables | Normoglycemia  (n = 2621) | |  | Prediabetes  (n = 3080) | | | T2DM  (n = 4192) | | |
| --- | --- | --- | --- | --- | --- | --- | --- | --- | --- |
|  | High  Group A1  (n = 806) | Low-moderate  Group B1  (n = 1815) | *p* value | High  Group A2  (n = 935) | Low-moderate  Group B2  (n = 2145) | *p* value | High  Group A3  (n = 1243) | Low-moderate  Group B3  (n = 2949) | *p* value |
| Age (years) | 59.4 ± 12.6 | 61.6 ± 12.9 | <0.001 | 62.0 ± 12.6 | 63.6 ± 12.4 | 0.001 | 62.8 ± 11.4 | 64.4 ± 11.7 | <0.001 |
| Male, n (%) | 686 (85.1) | 1436 (79.1) | <0.001 | 736 (78.7) | 1604 (74.8) | 0.019 | 928 (74.7) | 2058 (69.8) | 0.001 |
| LVEF (%) | 54.4 ± 10.3 | 52.7 ± 10.2 | <0.001 | 53.8 ± 10.7 | 52.8 ± 10.5 | 0.013 | 52.1 ± 11.2 | 51.3 ± 11.4 | 0.033 |
| BMI (kg/m^2^) | 24.3 ± 3.0 | 23.7 ± 2.9 | <0.001 | 24.5 ± 3.2 | 24.1 ± 3.1 | 0.004 | 24.7 ± 3.1 | 24.3 ± 3.1 | <0.001 |
| SBP (mmHg) | 134.4 ± 27.1 | 130.8 ± 27.7 | 0.002 | 132.0 ± 28.0 | 129.2 ± 26.4 | 0.010 | 133.0 ± 28.0 | 130.8 ± 27.3 | 0.020 |
| DBP (mmHg) | 83.0 ± 17.0 | 80.1 ± 16.2 | <0.001 | 80.1 ± 16.9 | 78.8 ± 15.9 | 0.052 | 79.8 ± 16.7 | 78.9 ± 15.7 | 0.103 |
| STEMI, n (%) | 462 (57.3) | 1081 (59.6) | 0.282 | 556 (59.5) | 1270 (59.2) | 0.893 | 681 (54.8) | 1520 (51.5) | 0.055 |
| Primary PCI, n (%) | 451 (97.6) | 1038 (96.0) | 0.118 | 538 (96.8) | 1222 (96.2) | 0.568 | 663 (97.4) | 1446 (95.1) | 0.016 |
| NSTEMI, n (%) | 344 (42.7) | 734 (40.4) | 0.282 | 379 (40.5) | 875 (40.8) | 0.893 | 562 (45.2) | 1429 (48.5) | 0.055 |
| PCI within 24 hours | 303 (88.1) | 652 (88.8) | 0.719 | 337 (88.9) | 750 (85.7) | 0.125 | 495 (88.1) | 1207 (84.5) | 0.039 |
| Cardiogenic shock, n (%) | 18 (2.2) | 78 (4.3) | 0.009 | 33 (3.5) | 93 (4.3) | 0.324 | 48 (3.9) | 126 (4.3) | 0.611 |
| Hypertension, n (%) | 293 (36.4) | 740 (40.8) | 0.033 | 403 (43.1) | 944 (44.0) | 0.641 | 705 (56.7) | 1821 (61.7) | 0.002 |
| Dyslipidemia, n (%) | 71 (8.8) | 163 (9.0) | 0.887 | 114 (12.2) | 259 (12.1) | 0.952 | 161 (13.0) | 461 (15.6) | 0.025 |
| Previous MI, n (%) | 20 (2.5) | 59 (3.3) | 0.323 | 31 (3.3) | 54 (2.5) | 0.214 | 50 (4.0) | 154 (5.2) | 0.116 |
| Previous PCI, n (%) | 30 (3.7) | 74 (4.1) | 0.745 | 47 (5.0) | 105 (4.9) | 0.857 | 83 (6.7) | 243 (8.2) | 0.088 |
| Previous CABG, n (%) | 3 (0.4) | 4 (0.2) | 0.446 | 1 (0.1) | 4 (0.2) | 0.614 | 8 (0.6) | 25 (0.8) | 0.570 |
| Previous HF, n (%) | 2 (0.2) | 10 (0.6) | 0.364 | 4 (0.4) | 22 (1.0) | 0.095 | 13 (1.0) | 42 (1.4) | 0.375 |
| Previous CVA, n (%) | 38 (4.7) | 75 (4.1) | 0.532 | 42 (4.5) | 115 (5.4) | 0.329 | 89 (7.2) | 241 (8.2) | 0.286 |
| Current smokers, n (%) | 393 (48.8) | 804 (44.3) | 0.034 | 473 (50.6) | 991 (46.2) | 0.025 | 531 (42.7) | 1123 (38.1) | 0.005 |
| Peak CK-MB (mg/dL) | 130.9 ± 151.6 | 137.6 ± 184.5 | 0.327 | 142.1 ± 172.4 | 145.5 ± 197.4 | 0.647 | 108.7 ± 151.3 | 101.6 ± 135.7 | 0.154 |
| Peak troponin-I (ng/mL) | 48.2 ± 75.3 | 48.7 ± 74.7 | 0.868 | 55.0 ± 89.0 | 45.4 ± 83.2 | 0.100 | 47.3 ± 91.7 | 47.6 ± 154.5 | 0.950 |
| NT-ProBNP (pg/mL) | 1466.6 ± 2739.2 | 1570.2 ± 3125.3 | 0.393 | 1474.5 ± 2226.2 | 1456.4 ± 2160.4 | 0.834 | 1997.4 ± 4332.4 | 2475.3 ± 6131.6 | 0.004 |
| hs-CRP (mg/dL) | 5.39 ± 10.7 | 7.32 ± 28.7 | 0.012 | 5.84 ± 13.3 | 10.2 ± 58.6 | 0.001 | 6.18 ± 18.8 | 10.9 ± 45.2 | <0.001 |
| Serum creatinine (mg/L) | 1.02 ± 1.23 | 0.99 ± 0.82 | 0.359 | 0.98 ± 0.69 | 1.00 ± 0.85 | 0.620 | 1.11 ± 1.02 | 1.21 ± 1.90 | 0.029 |
| eGFR (mL/min/1.73m2) | 93.6 ± 46.9 | 92.1 ± 34.2 | 0.440 | 88.6 ± 28.1 | 91.8 ± 44.4 | 0.014 | 88.2 ± 49.0 | 84.0 ± 37.8 | 0.007 |
| Blood glucose (mg/dL) | 135.1 ± 39.5 | 135.6 ± 48.0 | 0.785 | 145.9 ± 45.0 | 146.7 ± 49.0 | 0.672 | 227.5 ± 103.4 | 223.3 ± 97.8 | 0.228 |
| Hemoglobin A1C (%) | 5.4 ± 0.4 | 5.3 ± 0.4 | 0.160 | 6.0 ± 0.2 | 6.0 ± 0.2 | 0.513 | 7.8 ± 1.7 | 7.8 ± 3.0 | 0.625 |
| Total cholesterol (mg/dL) | 189.9 ± 40.2 | 179.2 ± 39.5 | <0.001 | 199.9 ± 44.3 | 186.2 ± 40.9 | <0.001 | 189.2 ± 53.9 | 176.7 ± 45.0 | <0.001 |
| Triglyceride (mg/L) | 126.6 ± 84.9 | 115.9 ± 89.2 | 0.003 | 151.2 ± 136.2 | 127.1 ± 90.0 | <0.001 | 157.4 ± 132.6 | 147.6 ± 123.1 | 0.026 |
| HDL-cholesterol (mg/L) | 44.7 ± 12.1 | 44.4 ± 15.5 | 0.548 | 44.2 ± 18.6 | 43.5 ± 13.2 | 0.353 | 42.1 ± 11.7 | 41.8 ± 13.5 | 0.445 |
| LDL-cholesterol (mg/L) | 123.3 ± 36.8 | 112.9 ± 34.5 | <0.001 | 130.6 ± 38.9 | 119.4 ± 50.7 | <0.001 | 119.2 ± 40.6 | 108.2 ± 35.9 | <0.001 |
| Discharge medications |  |  |  |  |  |  |  |  |  |
| Aspirin, n (%) | 802 (99.6) | 1806 (99.5) | 0.949 | 931 (99.6) | 2136 (99.6) | 0.990 | 1241 (99.8) | 2927 (99.3) | 0.023 |
| Clopidogrel, n (%) | 607 (75.3) | 1465 (80.7) | 0.002 | 764 (81.7) | 1832 (85.4) | 0.010 | 1000 (80.5) | 2570 (87.1) | <0.001 |
| Ticagrelor, n (%) | 144 (17.9) | 214 (11.8) | <0.001 | 118 (12.6) | 188 (8.8) | 0.001 | 159 (12.8) | 234 (7.9) | <0.001 |
| Prasugrel, n (%) | 51 (6.3) | 127 (7.0) | 0.557 | 49 (5.2) | 116 (5.4) | 0.931 | 82 (6.6) | 145 (4.9) | 0.028 |
| Cilostazole, n (%) | 113 (14.0) | 248 (13.7) | 0.807 | 168 (18.0) | 420 (19.6) | 0.319 | 225 (18.1) | 611 (20.7) | 0.053 |
| BBs, n (%) | 682 (84.6) | 1570 (86.5) | 0.200 | 811 (86.7) | 1845 (86.0) | 0.592 | 1085 (87.3) | 2572 (87.2) | 0.949 |
| ACEIs, n (%) | 419 (52.0) | 1161 (64.0) | <0.001 | 470 (50.3) | 1344 (62.7) | <0.001 | 579 (46.6) | 1669 (56.6) | <0.001 |
| ARBs, n (%) | 239 (29.7) | 397 (21.9) | <0.001 | 305 (32.6) | 463 (21.6) | <0.001 | 433 (34.8) | 856 (29.0) | <0.001 |
| CCBs, n (%) | 45 (5.6) | 93 (5.1) | 0.627 | 32 (3.4) | 135 (6.3) | 0.001 | 78 (6.3) | 235 (8.0) | 0.057 |
| Statin, n (%) |  |  |  |  |  |  |  |  |  |
| Atorvastatin, n (%) | 440 (54.6) | 835 (46.0) | <0.001 | 418 (44.7) | 987 (46.0) | 0.503 | 626 (50.4) | 1475 (50.0) | 0.838 |
| Rosuvastatin, n (%) | 312 (38.7) | 704 (38.8) | 0.970 | 440 (47.1) | 825 (38.5) | <0.001 | 501 (40.3) | 967 (32.8) | <0.001 |
| Simvastatin, n (%) | 6 (0.7) | 152 (8.4) | <0.001 | 7 (0.7) | 162 (7.6) | <0.001 | 8 (0.6) | 230 (7.8) | <0.001 |
| Pitavastatin, n (%) | 30 (3.7) | 98 (5.4) | 0.077 | 56 (6.0) | 147 (6.9) | 0.430 | 75 (6.0) | 237 (8.0) | 0.024 |
| Pravastatin, n (%) | 9 (1.1) | 25 (1.4) | 0.709 | 8 (0.9) | 24 (1.1) | 0.568 | 19 (1.5) | 39 (1.3) | 0.664 |
| Fluvastatin, n (%) | 9 (1.1) | 1 (0.1) | <0.001 | 6 (0.6) | 0 (0.0) | 0.001 | 14 (1.1) | 1 (0.0) | <0.001 |
| Diabetes management |  |  |  |  |  |  |  |  |  |
| Diet, n (%) |  |  |  |  |  |  | 120 (9.7) | 221 (7.5) | 0.022 |
| Oral agent, n (%) |  |  |  |  |  |  | 729 (58.6) | 1873 (63.5) | 0.003 |
| Insulin, n (%) |  |  |  |  |  |  | 64 (5.1) | 167 (5.7) | 0.553 |
| Untreated, n (%) | - |  |  |  |  |  | 330 (26.5) | 688 (23.3) | 0.008 |
| IRA |  |  |  |  |  |  |  |  |  |
| Left main, n (%) | 22 (2.7) | 25 (1.4) | 0.024 | 15 (1.6) | 30 (1.4) | 0.628 | 31 (2.5) | 50 (1.7) | 0.109 |
| LAD, n (%) | 392 (48.6) | 938 (51.7) | 0.158 | 453 (48.4) | 1072 (50.0) | 0.379 | 588 (47.3) | 1338 (45.4) | 0.222 |
| LCx, n (%) | 146 (18.1) | 290 (16.0) | 0.175 | 156 (16.7) | 360 (16.8) | 0.946 | 191 (15.4) | 504 (17.1) | 0.170 |
| RCA, n (%) | 246 (30.5) | 562 (31.0) | 0.821 | 311 (33.3) | 683 (31.8) | 0.438 | 433 (34.8) | 1057 (35.8) | 0.534 |
| Treated vessel |  |  |  |  |  |  |  |  |  |
| Left main, n (%) | 28 (3.5) | 43 (2.4) | 0.118 | 27 (2.9) | 55 (2.6) | 0.627 | 45 (3.6) | 84 (2.8) | 0.203 |
| LAD, n (%) | 471 (58.4) | 1092 (60.2) | 0.405 | 545 (58.3) | 1275 (59.4) | 0.550 | 744 (59.9) | 1716 (58.2) | 0.317 |
| LCx, n (%) | 216 (26.8) | 443 (26.4) | 0.193 | 256 (27.4) | 552 (25.7) | 0.340 | 365 (29.4) | 805 (27.3) | 0.173 |
| RCA, n (%) | 296 (36.7) | 659 (36.3) | 0.838 | 373 (39.9) | 827 (38.6) | 0.484 | 539 (43.4) | 1278 (43.3) | 0.988 |
| ACC/AHA lesion type |  |  |  |  |  |  |  |  |  |
| Type B1, n (%) | 98 (12.2) | 231 (12.7) | 0.685 | 115 (12.3) | 282 (13.1) | 0.519 | 140 (11.3) | 363 (12.3) | 0.341 |
| Type B2, n (%) | 272 (33.7) | 640 (35.3) | 0.452 | 292 (31.2) | 699 (32.6) | 0.458 | 415 (33.4) | 987 (33.5) | 0.959 |
| Type C, n (%) | 365 (45.3) | 837 (46.1) | 0.694 | 431 (46.1) | 974 (45.4) | 0.724 | 587 (47.2) | 1373 (46.6) | 0.693 |
| Extent of CAD |  |  |  |  |  |  |  |  |  |
| Single-vessel, n (%) | 449 (55.7) | 1003 (55.3) | 0.772 | 524 (56.0) | 1106 (51.6) | 0.017 | 569 (45.8) | 1244 (42.2) | 0.012 |
| Two-vessel, n (%) | 231 (28.7) | 546 (30.1) | 0.462 | 258 (27.6) | 679 (31.7) | 0.024 | 428 (34.4) | 975 (33.1) | 0.390 |
| ≥ Three-vessel, n (%) | 121 (15.0) | 266 (14.7) | 0.812 | 150 (16.0) | 360 (16.8) | 0.611 | 242 (19.5) | 730 (24.8) | <0.001 |
| Vascular access |  |  |  |  |  |  |  |  |  |
| Transradial, n (%) | 257 (31.9) | 547 (30.1) | 0.370 | 282 (30.2) | 627 (29.2) | 0.603 | 359 (28.9) | 836 (28.3) | 0.727 |
| Transfemoral, n (%) | 549 (68.1) | 1268 (69.9) | 0.370 | 653 (69.8) | 1518 (70.8) | 0.603 | 884 (71.1) | 2113 (71.7) | 0.727 |
| IVUS, n (%) | 209 (25.9) | 348 (19.2) | <0.001 | 265 (28.3) | 496 (23.1) | 0.002 | 328 (26.4) | 584 (19.8) | <0.001 |
| OCT, n (%) | 10 (1.2) | 11 (0.6) | 0.100 | 6 (0.6) | 22 (1.0) | 0.409 | 9 (0.7) | 22 (0.7) | 0.940 |
| FFR, n (%) | 8 (1.0) | 20 (1.1) | 0.802 | 15 (1.6) | 30 (1.4) | 0.628 | 17 (1.4) | 43 (1.5) | 0.888 |
| Drug-eluting stents^a^ |  |  |  |  |  |  |  |  |  |
| ZES, n (%) | 261 (32.4) | 541 (29.8) | 0.187 | 321 (34.3) | 739 (34.5) | 0.948 | 433 (34.8) | 993 (33.7) | 0.468 |
| EES, n (%) | 426 (52.9) | 944 (52.0) | 0.690 | 498 (53.3) | 1091 (50.9) | 0.220 | 649 (52.2) | 1539 (52.2) | 0.988 |
| BES, n (%) | 135 (16.7) | 322 (17.7) | 0.537 | 128 (13.7) | 307 (14.3) | 0.648 | 175 (14.1) | 384 (13.0) | 0.358 |
| Others, n (%) | 5 (0.6) | 46 (2.5) | 0.001 | 10 (1.1) | 55 (2.6) | 0.006 | 18 (1.4) | 89 (3.0) | 0.003 |
| Stent diameter (mm) | 3.17 ± 0.42 | 3.15 ± 0.42 | 0.268 | 3.16 ± 0.42 | 3.13 ± 0.41 | 0.060 | 3.14 ± 0.43 | 3.09 ± 0.41 | 0.001 |
| Stent length (mm) | 27.7 ± 11.2 | 27.4 ± 11.8 | 0.502 | 27.6 ± 12.7 | 27.0 ± 11.1 | 0.205 | 28.0 ± 12.5 | 27.8 ± 11.9 | 0.552 |
| Number of stent | 1.48 ± 0.80 | 1.40 ± 0.72 | 0.015 | 1.51 ± 0.84 | 1.47 ± 0.78 | 0.233 | 1.59 ± 0.87 | 1.54 ± 0.82 | 0.058 |

Values are means ± SD or numbers and percentages. The *p* values for continuous data obtained from the analysis of variance. The *p* values for categorical data from chi-square or Fisher’s exact test. LVEF: left ventricular ejection fraction; BMI: body mass index; SBP: systolic blood pressure; DBP: diastolic blood pressure; STEMI: ST-elevation myocardial infarction; NSTEMI: non-ST-elevation myocardial infarction; PCI: percutaneous coronary intervention; CABG: coronary artery bypass graft; HF: heart failure; CVA: cerebrovascular accident; CK-MB: creatine kinase myocardial band; NT-ProBNP: N-terminal pro-brain natriuretic peptide; hs-CRP: high sensitivity C-reactive protein; eGFR: estimated glomerular filtration rate; HDL: high-density lipoprotein; LDL: low-density lipoprotein; BBs: beta-blockers; ACEs: angiotensin converting enzyme inhibitors; ARBs: angiotensin receptor blockers; CCBs: calcium channel blockers; IRA: infarct-related artery; LAD: left anterior descending coronary artery; LCx: left circumflex coronary artery; RCA: right coronary artery; ACC/AHA: American College of Cardiology/American Heart Association; CAD: coronary artery disease; IVUS: intravascular ultrasound; OCT: optical coherence tomography; FFR: fractional flow reserve; ZES: zotarolimus-eluting stent; EES: everolimus-eluting stent; BES: biolimus-eluting stents.

^a^Drug-eluting stents were composed of ZES (Resolute Integrity stent; Medtronic, Inc., Minneapolis, MN), EES (Xience Prime stent, Abbott Vascular, Santa Clara, CA; or Promus Element stent, Boston Scientific, Natick, MA), BES (BioMatrix Flex stent, Biosensors International, Morges, Switzerland; or Nobori stent, Terumo Corporation, Tokyo, Japan), and others include any other newer-generation drug-eluting stents except for ZES, EES, and BES.

**Supplementary material 8** Comparison of baseline characteristics between statin users and statin non-users

| Variables | Normoglycemia  (n = 2,993) | |  | Prediabetes  (n = 3,588) | | | T2DM  (n = 5,031) | | |
| --- | --- | --- | --- | --- | --- | --- | --- | --- | --- |
|  | Statin users  Group A1+B1  (n = 2,621) | Statin nonusers  Group C1  (n = 372) | *p* value | Statin users  Group A2+B2  (n = 3,080) | Statin nonusers  Group C2  (n = 508) | *p* value | Statin users  Group A3+B3  (n = 4,192) | Statin nonusers  Group C3  (n = 839) | *p* value |
| Age (years) | 60.9 ± 12.9 | 63.1 ± 14.3 | 0.005 | 63.1 ± 12.5 | 64.8 ± 12.9 | 0.005 | 63.9 ± 11.6 | 65.7 ± 11.6 | <0.001 |
| Male, n (%) | 2122 (85.1) | 288 (77.4) | 0.108 | 2340 (78.7) | 372 (73.2) | 0.182 | 2986 (71.2) | 555 (66.2) | 0.003 |
| LVEF (%) | 53.2 ± 10.3 | 50.8 ± 13.0 | 0.001 | 53.1 ± 10.5 | 50.2 ± 13.0 | <0.001 | 51.5 ± 11.4 | 48.9 ± 13.0 | <0.001 |
| BMI (kg/m^2^) | 23.9 ± 3.0 | 23.5 ± 3.2 | 0.028 | 24.2 ± 3.2 | 23.7 ± 3.3 | 0.002 | 24.4 ± 3.1 | 24.1 ± 3.2 | 0.014 |
| SBP (mmHg) | 131.9 ± 27.5 | 125.2 ± 27.8 | <0.001 | 130.1 ± 26.9 | 128.8 ± 28.6 | 0.361 | 131.5 ± 27.5 | 128.0 ± 29.7 | 0.002 |
| DBP (mmHg) | 81.0 ± 16.5 | 78.0 ± 16.7 | 0.002 | 79.2 ± 16.2 | 78.6 ± 16.1 | 0.392 | 79.1 ± 16.0 | 76.7 ± 16.8 | <0.001 |
| STEMI, n (%) | 1543 (58.9) | 237 (63.7) | 0.075 | 1826 (59.3) | 324 (63.8) | 0.056 | 2201 (52.5) | 457 (54.5) | 0.298 |
| Primary PCI, n (%) | 1489 (96.5) | 227 (95.8) | 0.573 | 1760 (96.3) | 305 (94.1) | 0.063 | 2109 (95.8) | 439 (96.1) | 0.898 |
| NSTEMI, n (%) | 1078 (41.1) | 135 (36.3) | 0.075 | 1254 (40.7) | 184 (36.2) | 0.056 | 1991 (47.5) | 382 (45.5) | 0.298 |
| PCI within 24 hours | 955 (88.6) | 115 (85.2) | 0.257 | 1087 (86.7) | 148 (80.4) | 0.030 | 1702 (85.5) | 299 (78.3) | 0.001 |
| Cardiogenic shock, n (%) | 96 (3.7) | 22 (5.9) | 0.045 | 126 (4.1) | 26 (5.1) | 0.285 | 174 (4.2) | 65 (7.7) | <0.001 |
| Hypertension, n (%) | 1033 (39.4) | 171 (46.0) | 0.018 | 1347 (43.7) | 226 (44.5) | 0.751 | 2526 (60.3) | 508 (60.5) | 0.875 |
| Dyslipidemia, n (%) | 234 (8.9) | 14 (3.8) | <0.001 | 373 (12.1) | 44 (8.7) | 0.025 | 622 (13.0) | 93 (11.1) | 0.004 |
| Previous MI, n (%) | 79 (3.0) | 10 (2.7) | 0.871 | 85 (2.8) | 9 (1.8) | 0.231 | 204 (4.9) | 37 (4.4) | 0.658 |
| Previous PCI, n (%) | 104 (4.0) | 14 (3.8) | 0.850 | 152 (4.9) | 21 (4.1) | 0.503 | 326 (7.8) | 63 (7.5) | 0.832 |
| Previous CABG, n (%) | 7 (0.3) | 0 (0.0) | 0.318 | 5 (0.2) | 0 (0.0) | 0.363 | 33 (0.8) | 6 (0.7) | 0.828 |
| Previous HF, n (%) | 12 (0.5) | 2 (0.5) | 0.833 | 26 (0.8) | 9 (1.8) | 0.082 | 55 (1.3) | 24 (2.9) | 0.002 |
| Previous CVA, n (%) | 113 (4.3) | 28 (7.5) | 0.009 | 157 (5.1) | 25 (4.9) | 0.867 | 330 (7.9) | 67 (8.0) | 0.889 |
| Current smokers, n (%) | 1197 (45.7) | 167 (44.9) | 0.778 | 1464 (47.5) | 219 (43.1) | 0.064 | 1654 (39.5) | 297 (35.4) | 0.028 |
| Peak CK-MB (mg/dL) | 135.6 ± 175.0 | 173.9 ± 359.3 | 0.044 | 144.5 ± 204.8 | 164.0 ± 227.9 | 0.071 | 103.7 ± 140.5 | 111.7 ± 143.8 | 0.140 |
| Peak troponin-I (ng/mL) | 48.5 ± 74.9 | 50.2 ± 84.2 | 0.715 | 48.4 ± 96.4 | 50.1 ± 83.4 | 0.682 | 47.5 ± 138.9 | 48.8 ± 131.5 | 0.793 |
| NT-ProBNP (pg/mL) | 1538.4 ± 3011.7 | 2638.4 ± 4028.9 | <0.001 | 1461.9 ± 2180.2 | 2487.0 ± 3339.8 | <0.001 | 2333.6 ± 5661.8 | 3713.6 ± 5394.3 | <0.001 |
| hs-CRP (mg/dL) | 6.73 ± 24.6 | 10.8 ± 40.8 | 0.059 | 8.85 ± 49.5 | 13.7 ± 45.8 | 0.028 | 9.48 ± 39.4 | 19.3 ± 61.0 | <0.001 |
| Serum creatinine (mg/L) | 1.00 ± 0.97 | 1.04 ± 0.47 | 0.160 | 0.99 ± 0.81 | 1.17 ± 1.31 | 0.004 | 1.18 ± 1.69 | 1.37 ± 1.42 | 0.001 |
| eGFR (mL/min/1.73m2) | 92.6 ± 38.6 | 84.1 ± 33.8 | <0.001 | 90.9 ± 40.2 | 81.4 ± 30.4 | <0.001 | 85.3 ± 41.5 | 78.6 ± 51.7 | <0.001 |
| Blood glucose (mg/dL) | 135.4 ± 45.5 | 152.0 ± 73.8 | <0.001 | 146.5 ± 47.9 | 152.8 ± 53.1 | 0.013 | 224.6 ± 99.5 | 235.8 ± 105.8 | 0.005 |
| Hemoglobin A1C (%) | 5.3 ± 0.4 | 5.3 ± 0.5 | 0.448 | 6.0 ± 0.2 | 6.0 ± 0.2 | 0.143 | 7.8 ± 2.7 | 7.9 ± 3.5 | 0.388 |
| Total cholesterol (mg/dL) | 182.5 ± 40.0 | 172.0 ± 40.6 | <0.001 | 190.3 ± 42.4 | 184.4 ± 46.6 | 0.008 | 180.4 ± 48.2 | 170.1 ± 45.2 | <0.001 |
| Triglyceride (mg/L) | 119.2 ± 88.0 | 110.1 ± 76.8 | 0.037 | 134.4 ± 106.7 | 125.7 ± 85.0 | 0.039 | 150.5 ± 126.1 | 141.5 ± 125.7 | 0.059 |
| HDL-cholesterol (mg/L) | 44.5 ± 14.6 | 44.3 ± 19.6 | 0.827 | 43.7 ± 15.0 | 43.8 ± 17.8 | 0.928 | 41.9 ± 13.0 | 40.8 ± 18.1 | 0.107 |
| LDL-cholesterol (mg/L) | 116.1 ± 35.5 | 108.2 ± 37.2 | <0.001 | 122.8 ± 47.7 | 115.8 ± 37.6 | <0.001 | 111.5 ± 37.7 | 105.8 ± 39.8 | <0.001 |
| Discharge medications |  |  |  |  |  |  |  |  |  |
| Aspirin, n (%) | 2608 (99.5) | 357 (96.0) | 0.824 | 3067 (99.6) | 493 (97.0) | <0.001 | 4168 (99.4) | 801 (95.5) | <0.001 |
| Clopidogrel, n (%) | 2072 (79.1) | 331 (89.0) | <0.001 | 2596 (84.3) | 478 (94.1) | <0.001 | 3570 (85.2) | 770 (91.8) | <0.001 |
| Ticagrelor, n (%) | 358 (13.7) | 16 (4.3) | <0.001 | 306 (9.9) | 7 (1.4) | <0.001 | 393 (9.4) | 17 (2.0) | <0.001 |
| Prasugrel, n (%) | 178 (6.8) | 10 (2.7) | <0.001 | 165 (5.4) | 8 (1.6) | <0.001 | 227 (5.4) | 14 (1.7) | <0.001 |
| Cilostazole, n (%) | 361 (13.8) | 56 (15.1) | 0.522 | 588 (19.1) | 89 (17.5) | 0.402 | 836 (19.9) | 131 (15.6) | 0.003 |
| BBs, n (%) | 2252 (85.9) | 232 (62.4) | <0.001 | 2656 (86.2) | 308 (60.6) | <0.001 | 3657 (87.2) | 534 (63.6) | <0.001 |
| ACEIs, n (%) | 1580 (60.3) | 161 (43.3) | <0.001 | 1814 (58.9) | 194 (38.2) | <0.001 | 2248 (53.6) | 299 (35.6) | <0.001 |
| ARBs, n (%) | 636 (24.3) | 69 (18.5) | 0.016 | 768 (24.9) | 89 (17.5) | <0.001 | 1289 (30.7) | 212 (25.3) | 0.001 |
| CCBs, n (%) | 138 (5.3) | 28 (7.5) | 0.089 | 167 (5.4) | 29 (5.7) | 0.753 | 313 (7.5) | 57 (6.8) | 0.496 |
| Statin, n (%) |  |  |  |  |  |  |  |  |  |
| Atorvastatin, n (%) | 1275 (48.6) |  |  | 1405 (45.6) |  |  | 2101 (50.1) |  |  |
| Rosuvastatin, n (%) | 1016 (38.8) |  |  | 1265 (41.1) |  |  | 1468 (35.0) |  |  |
| Simvastatin, n (%) | 158 (6.0) |  |  | 169 (5.5) |  |  | 238 (5.7) |  |  |
| Pitavastatin, n (%) | 128 (4.9) |  |  | 203 (6.6) |  |  | 312 (7.4) |  |  |
| Pravastatin, n (%) | 34 (1.3) |  |  | 32 (1.0) |  |  | 58 (1.4) |  |  |
| Fluvastatin, n (%) | 10 (0.4) |  |  | 6 (0.2) |  |  | 15 (0.4) |  |  |
| Diabetes management |  |  |  |  |  |  |  |  |  |
| Diet, n (%) |  |  |  |  |  |  | 341 (8.1) | 66 (7.9) | 0.835 |
| Oral agent, n (%) |  |  |  |  |  |  | 2602 (62.1) | 545 (65.0) | 0.115 |
| Insulin, n (%) |  |  |  |  |  |  | 231 (5.5) | 50 (6.0) | 0.621 |
| Untreated, n (%) | - |  |  |  |  |  | 1018 (24.3) | 178 (21.2) | 0.089 |
| IRA |  |  |  |  |  |  |  |  |  |
| Left main, n (%) | 47 (1.8) | 7 (1.9) | 0.836 | 45 (1.5) | 9 (1.8) | 0.556 | 81 (1.9) | 20 (2.4) | 0.418 |
| LAD, n (%) | 1330 (50.7) | 179 (48.1) | 0.350 | 1525 (49.5) | 267 (52.6) | 0.271 | 1926 (45.9) | 398 (47.4) | 0.424 |
| LCx, n (%) | 436 (16.6) | 53 (14.2) | 0.244 | 516 (16.8) | 72 (14.2) | 0.155 | 695 (16.6) | 148 (17.6) | 0.453 |
| RCA, n (%) | 808 (30.8) | 133 (35.8) | 0.056 | 994 (32.3) | 160 (31.5) | 0.728 | 1490 (35.5) | 273 (32.5) | 0.096 |
| Treated vessel |  |  |  |  |  |  |  |  |  |
| Left main, n (%) | 71 (2.7) | 12 (3.2) | 0.611 | 82 (2.7) | 15 (3.0) | 0.708 | 129 (3.1) | 29 (3.5) | 0.587 |
| LAD, n (%) | 1563 (59.6) | 208 (55.9) | 0.172 | 1820 (59.1) | 294 (57.9) | 0.605 | 2460 (58.7) | 472 (56.3) | 0.193 |
| LCx, n (%) | 659 (25.1) | 77 (20.7) | 0.063 | 808 (26.2) | 112 (22.0) | 0.048 | 1170 (27.9) | 229 (27.3) | 0.716 |
| RCA, n (%) | 955 (36.4) | 149 (40.1) | 0.176 | 1200 (39.0) | 149 (35.2) | 0.115 | 1817 (43.3) | 330 (39.3) | 0.032 |
| ACC/AHA lesion type |  |  |  |  |  |  |  |  |  |
| Type B1, n (%) | 329 (12.6) | 63 (16.9) | 0.021 | 397 (12.9) | 84 (16.5) | 0.029 | 503 (12.0) | 135 (16.1) | 0.001 |
| Type B2, n (%) | 912 (34.8) | 88 (23.7) | <0.001 | 991 (32.2) | 153 (30.1) | 0.382 | 1402 (33.4) | 241 (28.7) | 0.008 |
| Type C, n (%) | 1202 (45.9) | 151 (40.6) | 0.058 | 1405 (45.6) | 181 (35.6) | <0.001 | 1960 (46.8) | 338 (40.3) | 0.001 |
| Extent of CAD |  |  |  |  |  |  |  |  |  |
| Single-vessel, n (%) | 1452 (55.4) | 194 (52.2) | 0.112 | 1630 (52.9) | 251 (49.4) | 0.214 | 1813 (43.2) | 354 (42.2) | 0.589 |
| Two-vessel, n (%) | 777 (29.6) | 112 (30.1) | 0.855 | 937 (30.4) | 156 (30.7) | 0.917 | 1403 (33.5) | 258 (30.8) | 0.137 |
| ≥ Three-vessel, n (%) | 387 (14.8) | 66 (17.7) | 0.134 | 510 (16.6) | 101 (19.9) | 0.074 | 972 (23.2) | 227 (27.1) | 0.019 |
| Vascular access |  |  |  |  |  |  |  |  |  |
| Transradial, n (%) | 804 (30.7) | 111 (29.8) | 0.743 | 909 (29.5) | 147 (28.9) | 0.792 | 1195 (28.5) | 221 (26.3) | 0.203 |
| Transfemoral, n (%) | 1817 (69.3) | 261 (70.2) | 0.743 | 2171 (70.5) | 361 (71.1) | 0.792 | 2997 (71.5) | 618 (73.7) | 0.203 |
| IVUS, n (%) | 557 (21.3) | 88 (23.7) | 0.291 | 761 (24.7) | 98 (19.3) | 0.008 | 912 (21.8) | 161 (19.2) | 0.106 |
| OCT, n (%) | 21 (0.8) | 2 (0.5) | 0.586 | 28 (0.9) | 2 (0.4) | 0.302 | 31 (0.7) | 5 (0.6) | 0.823 |
| FFR, n (%) | 28 (1.1) | 1 (0.3) | 0.250 | 45 (1.5) | 0 (0.0) | 0.002 | 60 (1.4) | 2 (0.2) | 0.002 |
| Drug-eluting stents^+^ |  |  |  |  |  |  |  |  |  |
| ZES, n (%) | 802 (30.6) | 141 (37.9) | 0.005 | 1060 (34.4) | 193 (38.0) | 0.117 | 1426 (34.0) | 317 (37.8) | 0.039 |
| EES, n (%) | 1370 (52.3) | 188 (50.5) | 0.531 | 1589 (51.6) | 266 (52.4) | 0.747 | 2188 (52.2) | 410 (48.9) | 0.078 |
| BES, n (%) | 457 (17.4) | 37 (9.9) | <0.001 | 435 (14.1) | 41 (8.1) | <0.001 | 559 (13.3) | 97 (11.6) | 0.178 |
| Others, n (%) | 51 (1.9) | 10 (2.7) | 0.327 | 65 (2.1) | 10 (2.0) | 0.836 | 107 (2.6) | 24 (2.9) | 0.634 |
| Stent diameter (mm) | 3.15 ± 0.42 | 3.21 ± 0.42 | 0.013 | 3.14 ± 0.41 | 3.15 ± 0.44 | 0.747 | 3.10 ± 0.41 | 3.07 ± 0.42 | 0.028 |
| Stent length (mm) | 27.5 ± 11.6 | 25.4 ± 8.99 | <0.001 | 27.2 ± 11.6 | 24.9 ± 7.61 | <0.001 | 27.8 ± 12.0 | 25.7 ± 10.0 | <0.001 |
| Number of stent | 1.43 ± 0.74 | 1.41 ± 0.74 | 0.709 | 1.49 ± 0.80 | 1.42 ± 0.74 | 0.047 | 1.56 ± 0.83 | 1.51 ± 0.78 | 0.115 |

Values are means ± SD or numbers and percentages. The *p* values for continuous data obtained from the analysis of variance. The *p* values for categorical data from chi-square or Fisher’s exact test. LVEF: left ventricular ejection fraction; BMI: body mass index; SBP: systolic blood pressure; DBP: diastolic blood pressure; STEMI: ST-elevation myocardial infarction; NSTEMI: non-ST-elevation myocardial infarction; PCI: percutaneous coronary intervention; CABG: coronary artery bypass graft; HF: heart failure; CVA: cerebrovascular accident; CK-MB: creatine kinase myocardial band; NT-ProBNP: N-terminal pro-brain natriuretic peptide; hs-CRP: high sensitivity C-reactive protein; eGFR: estimated glomerular filtration rate; HDL: high-density lipoprotein; LDL: low-density lipoprotein; BBs: beta-blockers; ACEs: angiotensin converting enzyme inhibitors; ARBs: angiotensin receptor blockers; CCBs: calcium channel blockers; IRA: infarct-related artery; LAD: left anterior descending coronary artery; LCx: left circumflex coronary artery; RCA: right coronary artery; ACC/AHA: American College of Cardiology/American Heart Association; CAD: coronary artery disease; IVUS: intravascular ultrasound; OCT: optical coherence tomography; FFR: fractional flow reserve; ZES: zotarolimus-eluting stent; EES: everolimus-eluting stent; BES: biolimus-eluting stents.

^a^Drug-eluting stents were composed of ZES (Resolute Integrity stent; Medtronic, Inc., Minneapolis, MN), EES (Xience Prime stent, Abbott Vascular, Santa Clara, CA; or Promus Element stent, Boston Scientific, Natick, MA), BES (BioMatrix Flex stent, Biosensors International, Morges, Switzerland; or Nobori stent, Terumo Corporation, Tokyo, Japan), and others include any other newer-generation drug-eluting stents except for ZES, EES, and BES.

**Supplementary material 9** Comparison of baseline characteristics between high-intensity and low-moderate-intensity statin treatment or between statin users and nonusers.

| Variables | High-intensity  (n = 2,984) | Low-moderate-intensity (n = 6,909) | *p* value | Statin (+)  (n = 9,893) | Statin (-)  (n = 1,719) | *p* value |
| --- | --- | --- | --- | --- | --- | --- |
| Age (years) | 61.6 ± 12.2 | 63.4 ± 12.3 | <0.001 | 62.9 ± 12.3 | 64.9 ± 12.6 | <0.001 |
| Men, n (%) | 2350 (78.8) | 5098 (73.8) | <0.001 | 7448 (75.3) | 1215 (70.7) | <0.001 |
| LVEF (%) | 53.3 ± 10.9 | 52.1 ± 10.8 | <0.001 | 52.5 ± 10.9 | 49.7 ± 13.1 | <0.001 |
| BMI (kg/m^2^) | 24.5 ± 3.1 | 24.1 ± 3.1 | <0.001 | 24.2 ± 3.1 | 23.9 ± 3.2 | <0.001 |
| SBP (mmHg) | 133.1 ± 27.8 | 130.3 ± 27.1 | <0.001 | 131.2 ± 27.4 | 127.6 ± 29.0 | <0.001 |
| DBP (mmHg) | 80.7 ± 16.9 | 79.2 ± 15.9 | <0.001 | 79.6 ± 16.2 | 77.5 ± 16.6 | <0.001 |
| STEMI, n (%) | 1699 (56.9) | 3871 (56.0) | 0.403 | 5570 (56.3) | 1018 (59.2) | 0.024 |
| Primary PCI, n (%) | 1652 (97.2) | 3706 (95.7) | 0.007 | 5358 (96.2) | 971 (95.4) | 0.221 |
| NSTEMI, n (%) | 1285 (43.1) | 3038 (44.0) | 0.403 | 4323 (43.7) | 701 (40.8) | 0.024 |
| PCI within 24 hours | 1135 (88.3) | 2609 (85.9) | 0.031 | 3744 (86.6) | 562 (80.2) | <0.001 |
| Cardiogenic shock, n (%) | 99 (3.3) | 297 (4.3) | 0.022 | 396 (4.0) | 113 (6.6) | <0.001 |
| Hypertension, n (%) | 1401 (47.0) | 3505 (50.7) | 0.001 | 4906 (49.6) | 905 (52.6) | 0.019 |
| Dyslipidemia, n (%) | 346 (11.6) | 883 (12.8) | 0.104 | 1229 (12.4) | 151 (8.9) | <0.001 |
| Previous MI, n (%) | 101 (3.4) | 267 (3.9) | 0.271 | 368 (3.7) | 56 (3.3) | 0.365 |
| Previous PCI, n (%) | 160 (5.4) | 422 (6.1) | 0.162 | 582 (5.9) | 98 (5.7) | 0.767 |
| Previous CABG, n (%) | 12 (0.4) | 33 (0.5) | 0.745 | 45 (0.5) | 6 (0.3) | 0.693 |
| Previous HF, n (%) | 19 (0.6) | 74 (1.1) | 0.041 | 93 (0.9) | 35 (2.0) | <0.001 |
| Previous CVA, n (%) | 169 (5.7) | 431 (6.2) | 0.272 | 600 (6.1) | 120 (7.0) | 0.146 |
| Current smokers, n (%) | 1397 (46.8) | 2918 (42.2) | <0.001 | 4315 (43.6) | 683 (39.7) | 0.003 |
| Peak CK-MB (mg/dL) | 125.2 ± 158.9 | 124.7 ± 178.5 | 0.897 | 124.8 ± 172.8 | 140.6 ± 232.6 | 0.007 |
| Peak troponin-I (ng/mL) | 50.0 ± 118.3 | 47.2 ± 117.5 | 0.287 | 48.0 ± 102.8 | 49.5 ± 109.6 | 0.614 |
| NT-ProBNP (pg/mL) | 1690.2 ± 3385.1 | 1921.2 ± 4504.3 | 0.005 | 1851.5 ± 4199.4 | 3118.4 ± 4931.1 | <0.001 |
| hs-CRP (mg/dL) | 5.9 ± 15.3 | 9.7 ± 46.5 | <0.001 | 8.6 ± 39.8 | 15.8 ± 52.9 | <0.001 |
| Serum creatinine (mg/L) | 1.05 ± 1.00 | 1.09 ± 1.40 | 0.141 | 1.08 ± 1.29 | 1.24 ± 1.25 | <0.001 |
| eGFR (mL/min/1.73m^2^) | 89.8 ± 43.0 | 88.6 ± 39.3 | 0.196 | 89.0 ± 40.5 | 80.6 ± 42.7 | <0.001 |
| blood glucose (mg/dL) | 177.3 ± 85.9 | 176.8 ± 84.4 | 0.784 | 176.9 ± 84.8 | 193.0 ± 95.9 | <0.001 |
| Hemoglobin A1c (%) | 6.57 ± 1.56 | 6.57 ± 2.25 | 0.989 | 6.6 ± 2.0 | 6.8 ± 2.7 | 0.003 |
| Total cholesterol (mg/dL) | 192.7 ± 47.8 | 180.3 ± 42.6 | <0.001 | 184.0 ± 44.6 | 174.8 ± 45.1 | <0.001 |
| Triglyceride (mg/L) | 147.2 ± 123.5 | 132.9 ± 106.1 | <0.001 | 137.2 ± 111.8 | 130.0 ± 106.2 | 0.010 |
| HDL cholesterol (mg/L) | 43.5 ± 14.3 | 43.0 ± 14.0 | 0.156 | 43.2 ± 14.1 | 42.5 ± 18.4 | 0.134 |
| LDL cholesterol (mg/L) | 123.9 ± 39.4 | 112.9 ± 41.0 | <0.001 | 116.2 ± 40.8 | 109.3 ± 38.8 | <0.001 |
| Discharge medications, n (%) |  |  |  |  |  |  |
| Aspirin, n (%) | 2974 (99.7) | 6869 (99.4) | 0.041 | 9843 (99.5) | 1651 (98.0) | <0.001 |
| Clopidogrel, n (%) | 2371 (79.5) | 5845 (84.6) | <0.001 | 8238 (83.3) | 1579 (91.9) | <0.001 |
| Ticagrelor, n (%) | 421 (14.1) | 636 (9.2) | <0.001 | 1057 (10.7) | 40 (2.3) | <0.001 |
| Prasugrel, n (%) | 182 (6.1) | 388 (5.6) | 0.344 | 570 (5.8) | 32 (1.9) | <0.001 |
| Cilostazole, n (%) | 506 (17.0) | 1279 (18.5) | 0.068 | 1785 (18.0) | 276 (16.1) | 0.047 |
| BBs, n (%) | 2578 (86.4) | 5897 (86.7) | 0.724 | 8565 (86.6) | 1074 (62.5) | <0.001 |
| ACEIs, n (%) | 1468 (49.2) | 4174 (60.4) | <0.001 | 5642 (57.0) | 654 (38.0) | <0.001 |
| ARBs, n (%) | 977 (32.7) | 1716 (24.8) | <0.001 | 2693 (27.2) | 370 (21.5) | <0.001 |
| CCBs, n (%) | 155 (5.2) | 463 (6.7) | 0.004 | 618 (6.2) | 114 (6.6) | 0.557 |
| Statin, n (%) |  |  |  |  |  |  |
| Atorvastatin, n (%) | 1484 (49.7) | 3297 (47.7) | 0.066 | 4781 (48.3) |  |  |
| Rosuvastatin, n (%) | 1253 (42.0) | 2496 (36.1) | <0.001 | 3749 (37.9) |  |  |
| Simvastatin, n (%) | 21 (0.7) | 544 (7.9) | <0.001 | 565 (5.7) |  |  |
| Pitavastatin, n (%) | 161 (5.4) | 482 (7.0) | 0.003 | 643 (6.5) |  |  |
| Pravastatin, n (%) | 36 (1.2) | 88 (1.3) | 0.844 | 124 (1.3) |  |  |
| Fluvastatin, n (%) | 29 (1.0) | 2 (0.0) | <0.001 | 31 (0.3) |  |  |
| Diabetes management n (%) |  |  |  |  |  |  |
| Diet, n (%) | 120/1234 (9.7) | 221/2949 (7.5) | 0.019 | 341/4192 (8.1) | 66/839 (7.9) | 0.835 |
| Oral agent, n (%) | 729/1243 (58.6) | 1873/2949 (63.6) | 0.003 | 2602/4192 (62.1) | 545/839 (65.0) | 0.118 |
| Insulin, n (%) | 64/1243 (5.1) | 167/2949 (5.7) | 0.553 | 231/4192 (5.5) | 50/839 (6.0) | 0.621 |
| Untreated, n (%) | 330/1243 (26.5) | 688/2949 (23.3) | 0.004 | 1018/4192 (24.3) | 178/839 (21.1) | 0.082 |
| IRA |  |  |  |  |  |  |
| Left main, n (%) | 68 (2.3) | 105 (1.5) | 0.010 | 173 (1.7) | 36 (2.1) | 0.327 |
| LAD, n (%) | 1433 (48.0) | 3348 (48.5) | 0.442 | 4781 (48.3) | 844 (49.1) | 0.398 |
| LCx, n (%) | 493 (16.5) | 1154 (16.7) | 0.837 | 1647 (16.6) | 273 (15.9) | 0.430 |
| RCA, n (%) | 990 (33.2) | 2302 (33.3) | 0.891 | 3292 (33.3) | 566 (32.9) | 0.776 |
| Treated vessel |  |  |  |  |  |  |
| Left main, n (%) | 100 (3.4) | 182 (2.6) | 0.056 | 282 (2.9) | 56 (3.3) | 0.352 |
| LAD, n (%) | 1760 (59.0) | 4083 (59.1) | 0.915 | 5843 (59.1) | 974 (56.7) | 0.063 |
| LCx, n (%) | 837 (28.0) | 1800 (26.1) | 0.040 | 2637 (26.7) | 418 (24.3) | 0.042 |
| RCA, n (%) | 1208 (40.5) | 2764 (40.0) | 0.657 | 3972 (40.1) | 658 (38.3) | 0.144 |
| ACC/AHA lesion type |  |  |  |  |  |  |
| Type B1, n (%) | 353 (11.8) | 876 (12.7) | 0.240 | 1229 (12.4) | 282 (16.4) | <0.001 |
| Type B2, n (%) | 979 (32.8) | 2326 (33.7) | 0.406 | 3305 (33.4) | 482 (28.0) | <0.001 |
| Type C, n (%) | 1383 (46.3) | 3184 (46.1) | 0.810 | 4567 (46.2) | 670 (39.0) | <0.001 |
| Extent of CAD |  |  |  |  |  |  |
| Single-vessel, n (%) | 1542 (51.7) | 3327 (48.2) | 0.001 | 4895 (49.5) | 782 (45.5) | 0.001 |
| Two-vessel, n (%) | 917 (30.7) | 2200 (31.8) | 0.275 | 3117 (31.5) | 526 (30.6) | 0.454 |
| ≥ Three-vessel, n (%) | 513 (17.2) | 1356 (19.6) | 0.005 | 1869 (18.9) | 394 (22.9) | <0.001 |
| Vascular access |  |  |  |  |  |  |
| Transradial, n (%) | 898 (30.1) | 2010 (29.1) | 0.316 | 2908 (29.4) | 479 (27.9) | 0.198 |
| Transfemoral, n (%) | 2086 (69.9) | 4899 (70.9) | 0.316 | 6985 (70.6) | 1240 (72.1) | 0.198 |
| IVUS, n (%) | 802 (26.9) | 1428 (20.7) | <0.001 | 2230 (22.5) | 347 (20.2) | 0.030 |
| OCT, n (%) | 25 (0.8) | 55 (0.8) | 0.808 | 80 (0.8) | 9 (0.5) | 0.292 |
| FFR, n (%) | 40 (1.3) | 93 (1.3) | 0.982 | 133 (1.3) | 3 (0.2) | <0.001 |
| Drug-eluting stents^a^ |  |  |  |  |  |  |
| ZES, n (%) | 1015 (34.0) | 2273 (32.9) | 0.280 | 3288 (33.2) | 651 (37.9) | <0.001 |
| EES, n (%) | 1573 (52.7) | 3574 (51.7) | 0.368 | 5147 (52.0) | 864 (50.3) | 0.176 |
| BES, n (%) | 438 (14.7) | 1013 (14.7) | 0.983 | 1451 (14.7) | 175 (10.2) | <0.001 |
| Others, n (%) | 33 (1.1) | 190 (2.8) | <0.001 | 223 (2.3) | 44 (2.6) | 0.436 |
| Stent diameter (mm) | 3.15 ± 0.43 | 3.12 ± 0.41 | <0.001 | 3.13 ± 0.42 | 3.12 ± 0.43 | 0.634 |
| Stent length (mm) | 27.8 ± 12.2 | 27.4 ± 11.6 | 0.154 | 27.5 ± 11.8 | 25.4 ± 9.14 | <0.001 |
| Number of stents | 1.54 ± 0.84 | 1.48 ± 0.78 | 0.002 | 1.50 ± 0.80 | 1.46 ± 0.76 | 0.046 |

Values are means ± SD or numbers and percentages. The *p* values for continuous data obtained from the analysis of variance. The *p* values for categorical data from chi-square or Fisher’s exact test. LVEF: left ventricular ejection fraction; BMI: body mass index; SBP: systolic blood pressure; DBP: diastolic blood pressure; STEMI: ST-elevation myocardial infarction; NSTEMI: non-ST-elevation myocardial infarction; PCI: percutaneous coronary intervention; CABG: coronary artery bypass graft; HF: heart failure; CVA: cerebrovascular accident; CK-MB: creatine kinase myocardial band; NT-ProBNP: N-terminal pro-brain natriuretic peptide; hs-CRP: high sensitivity C-reactive protein; eGFR: estimated glomerular filtration rate; HDL: high-density lipoprotein; LDL: low-density lipoprotein; BBs: beta-blockers; ACEs: angiotensin converting enzyme inhibitors; ARBs: angiotensin receptor blockers; CCBs: calcium channel blockers; IRA: infarct-related artery; LAD: left anterior descending coronary artery; LCx: left circumflex coronary artery; RCA: right coronary artery; ACC/AHA: American College of Cardiology/American Heart Association; CAD: coronary artery disease; IVUS: intravascular ultrasound; OCT: optical coherence tomography; FFR: fractional flow reserve; ZES: zotarolimus-eluting stent; EES: everolimus-eluting stent; BES: biolimus-eluting stents.

^a^Drug-eluting stents were composed of ZES (Resolute Integrity stent; Medtronic, Inc., Minneapolis, MN), EES (Xience Prime stent, Abbott Vascular, Santa Clara, CA; or Promus Element stent, Boston Scientific, Natick, MA), BES (BioMatrix Flex stent, Biosensors International, Morges, Switzerland; or Nobori stent, Terumo Corporation, Tokyo, Japan), and others include any other newer-generation drug-eluting stents except for ZES, EES, and BES.

**Supplementary material 10** Clinical outcomes between statin users and non-users at 2 years

| Outcomes | Statin (+)  (n = 2621) | Statin (-)  (n = 372) | Log-rank | Unadjusted | | Adjusted^a^ | |
| --- | --- | --- | --- | --- | --- | --- | --- |
|  |  |  |  | HR (95% CI) | *p* value | HR (95% CI) | *p* value |
| Normoglycemia | Group A1+B1 | Group C1 |  |  |  |  |  |
| MACE | 127 (5.5) | 51 (14.0) | <0.001 | 2.802 (2.024-3.878) | <0.001 | 2.082 (1.456-2.977) | <0.001 |
| All-cause death | 36 (1.5) | 34 (9.3) | <0.001 | 6.626 (4.145-10.59) | <0.001 | 3.109 (1.815-5.326) | <0.001 |
| Cardiac death | 25 (1.0) | 26 (7.1) | <0.001 | 7.358 (4.248-12.75) | <0.001 | 3.158 (1.667-5.982) | <0.001 |
| Re-MI | 36 (1.6) | 6 (1.8) | 0.758 | 1.146 (0.483-2.720) | 0.758 | 1.183 (0.478-2.929) | 0.716 |
| Any revascularization | 68 (3.1) | 11 (3.3) | 0.766 | 1.101 (0.582-2.083) | 0.766 | 1.062 (0.547-2.060) | 0.859 |
|  | Statin (+)  (n = 3080) | Statin (-)  (n = 508) | Log-rank | Unadjusted | | Adjusted^a^ | |
|  |  |  |  | HR (95% CI) | *p* value | HR (95% CI) | *p* value |
| Prediabetes | Group A2+B2 | Group C2 |  |  |  |  |  |
| MACE | 200 (7.0) | 79 (15.8) | <0.001 | 2.455 (1.884-3.173) | <0.001 | 1.813 (1.363-2.413) | <0.001 |
| All-cause death | 69 (2.4) | 57 (11.3) | <0.001 | 5.119 (3.604-7.271) | < 0.001 | 2.426 (1.631-3.608) | <0.001 |
| Cardiac death | 49 (1.7) | 51 (10.1) | <0.001 | 6.457 (4.362-9.557) | < 0.001 | 3.011 (1.938-4.677) | <0.001 |
| Re-MI | 51 (1.8) | 10 (2.1) | 0.580 | 1.211 (0.615-2.385) | 0.580 | 1.178 (0.575-2.413) | 0.654 |
| Any revascularization | 88 (3.2) | 22 (4.9) | 0.065 | 1.548 (0.970-2.470) | 0.067 | 1.554 (0.956-2.526) | 0.075 |
|  | Statin (+)  (n = 4192) | Statin (-)  (n = 839) | Log-rank | Unadjusted | | Adjusted^a^ | |
|  |  |  |  | HR (95% CI) | *p* value | HR (95% CI) | *p* value |
| T2DM | Group A3+B3 | Group C3 |  |  |  |  |  |
| MACE | 350 (9.2) | 153 (18.5) | <0.001 | 2.247 (1.858-2.718) | <0.001 | 1.775 (1.455-2.165) | <0.001 |
| All-cause death | 140 (3.6) | 113 (13.6) | <0.001 | 4.162 (3.248-5.333) | <0.001 | 2.676 (2.061-3.475) | <0.001 |
| Cardiac death | 91 (2.3) | 94 (11.3) | <0.001 | 5.323 (3.990-7.103) | <0.001 | 3.173 (2.341-4.301) | <0.001 |
| Re-MI | 98 (2.7) | 24 (3.2) | 0.348 | 1.238 (0.792-1.935) | 0.349 | 1.173 (0.742-1.852) | 0.494 |
| Any revascularization | 144 (3.8) | 48 (6.6) | 0.001 | 1.715 (1.237-2.378) | 0.001 | 1.637 (1.171-2.290) | 0.004 |
|  | Statin (+)  (n = 9893) | Statin (-)  (n = 1719) | Log-rank | Unadjusted | | Adjusted^a^ | |
|  |  |  |  | HR (95% CI) | *p* value | HR (95% CI) | *p* value |
| Total | Group A1+A2+A3+  B1+B2+B3 | Group C1+C2+ C3 |  |  |  |  |  |
| MACE | 677 (7.5) | 283 (16.7) | <0.001 | 2.448 (2.130-2.812) | <0.001 | 2.104 (1.820-2.431) | <0.001 |
| All-cause death | 245 (2.7) | 204 (12.0) | <0.001 | 4.888 (4.059-5.887) | <0.001 | 3.498 (2.876-4.255) | <0.001 |
| Cardiac death | 165 (1.8) | 171 (10.1) | <0.001 | 6.097 (4.922-7.551) | <0.001 | 4.171 (3.329-5.226) | <0.001 |
| Re-MI | 185 (2.1) | 40 (2.6) | 0.204 | 1.248 (0.886-1.756) | 0.205 | 1.180 (0.829-1.679) | 0.359 |
| Any revascularization | 300 (3.4) | 81 (5.4) | <0.001 | 1.573 (1.230-2.010) | <0.001 | 1.497 (1.163-1.927) | 0.002 |

^a^ Adjusted by age, male, LVEF, BMI, SBP, DBP, cardiogenic shock, previous heart failure, NT-ProBNP, serum creatinine, eGFR, blood glucose, total cholesterol, LDL-cholesterol, aspirin, clopidogrel, ticagrelor, BB, ACEI, ACC/AHA type B2 lesion, single vessel disease, ≥ Three-vessel disease, and stent length.

HR: hazard ratio; CI: confidence interval; T2DM: type 2 diabetes mellitus; MACE: major adverse cardiac events; Re-MI: recurrent myocardial infarction; LVEF: left ventricular ejection fraction; BMI: body mass index; SBP: systolic blood pressure; DBP: diastolic blood pressure; NT-ProBNP: N-terminal pro-brain natriuretic peptide; eGFR: estimated glomerular filtration rate; LDL: low-density lipoprotein; BB: beta-blockers; ACEI: angiotensin converting enzyme inhibitors; ACC/AHA: American College of Cardiology/American Heart Association.

**Supplementary material 11** Multivariate Cox-proportional regression analysis for independent predictor of MACE in high-intensity statin users

| Variables | Unadjusted |  | Adjusted |  |
| --- | --- | --- | --- | --- |
|  | HR (95% CI) | *p* value | HR (95% CI) | *p* value |
| Group |  |  |  |  |
| Group A1 vs. Group A2 | 2.262 (1.400 - 3.655) | 0.001 | 2.253 (1.383 - 3.671) | 0.001 |
| Group A1 vs. Group A3 | 2.651 (1.682 - 4.179) | <0.001 | 2.267 (1.419 - 3.621) | 0.001 |
| Group A2 vs. Group A3 | 1.178 (0.855 - 1.624) | 0.316 | 1.032 (0.740 - 1.438) | 0.853 |
| Age ≥ 65 years | 1.276 (0.952 - 1.710) | 0.103 | 1.078 (0.774 - 1.502) | 0.656 |
| Male | 1.171 (0.831 - 1.649) | 0.367 | 1.028 (0.702 - 1.507) | 0.886 |
| LVEF <40% | 1.973 (1.358 - 2.865) | <0.001 | 1.758 (1.168 - 2.646) | 0.007 |
| STEMI | 1.331 (0.993 - 1.782) | 0.055 | 1.113 (0.818 - 1.514) | 0.495 |
| Cardiogenic shock | 1.682 (1.328 - 2.531) | <0.001 | 1.356 (1.129 - 2.087) | 0.002 |
| Hypertension | 1.390 (1.036 - 1.865) | 0.028 | 1.264 (0.926 - 1.726) | 0.140 |
| Dyslipidemia | 1.038 (0.652 - 1.652) | 0.876 | 1.023 (0.637 - 1.644) | 0.924 |
| Current smokers | 1.417 (1.050 - 1.913) | 0.023 | 1.194 (0.854 - 1.668) | 0.300 |
| NT-ProBNP | 1.001 (1.000 - 1.002) | <0.001 | 1.000 (0.999 - 1.001) | 0.011 |
| ACEI | 1.423 (1.059 - 1.912) | 0.019 | 1.218 (0.893 - 1.661) | 0.212 |
| Atorvastatin | 1.612 (1.203 - 2.428) | 0.009 | 1.578 (1.008 - 2.472) | 0.046 |
| Rosuvastatin | 1.232 (0.909 - 1.670) | 0.178 | 1.883 (1.195 - 2.967) | 0.006 |
| Simvastatin | 2.308 (0.737 - 7.225) | 0.151 | 1.397 (0.421 - 4.639) | 0.626 |
| eGFR < 60mL/min/1.73m^2^ | 1.524 (1.073 - 2.166) | 0.014 | 1.687 (1.267 - 3.807) | 0.003 |
| ACC/AHA type B2 lesion | 2.010 (1.073 - 2.166) | <0.001 | 1.879 (1.388 - 2.545) | <0.001 |
| Multivessel disease | 1.402 (1.501 - 2.693) | 0.025 | 1.098 (0.783 - 1.540) | 0.588 |
| Stent diameter < 3.0mm | 1.093 (0.784 - 1.524) | 0.600 | 1.139 (0.811 - 1.455) | 0.452 |
| Number of stent | 1.298 (1.130 - 1.490) | <0.001 | 1.233 (1.046 - 1.455) | 0.013 |

MACE: major adverse cardiac events; HR: hazard ratio; CI: confidence interval; Group A1: high-intensity statin/normoglycemia; Group A2: high-intensity statin/prediabetes; Group A3: high-intensity statin/T2DM; LVEF: left ventricular ejection fraction; STEMI: ST-segment elevation myocardial infarction; NT-ProBNP: N-terminal pro-brain natriuretic peptide; ACEI: angiotensin converting enzyme inhibitors; eGFR: estimated glomerular filtration rate; ACC/AHA: American College of Cardiology/American Heart Association.

**Supplementary material 12** Multivariate Cox-proportional regression analysis for independent predictor of MACE in low-moderate-intensity statin users

| Variables | Unadjusted |  | Adjusted |  |
| --- | --- | --- | --- | --- |
|  | HR (95% CI) | *p* value | HR (95% CI) | *p* value |
| Group |  |  |  |  |
| Group B1 vs. Group B2 | 1.089 (0.845 - 1.405) | 0.509 | 1.060 (0.820 - 1.370) | 0.657 |
| Group B1 vs. Group B3 | 1.463 (1.164 - 1.837) | 0.001 | 1.260 (1.030 - 1.594) | 0.025 |
| Group B2 vs. Group B3 | 1.347 (1.096 - 1.657) | 0.005 | 1.089 (0.878 - 1.350) | 0.439 |
| Age ≥ 65 years | 1.467 (1.229 - 1.751) | <0.001 | 1.140 (0.932 - 1.396) | 0.203 |
| Male | 1.491 (1.238 - 1.794) | <0.001 | 1.406 (1.133 - 1.746) | 0.002 |
| LVEF <40% | 2.194 (1.769 - 2.721) | <0.001 | 1.895 (1.516 - 2.369) | <0.001 |
| STEMI | 1.249 (1.048 - 1.489) | 0.013 | 1.161 (0.966 - 1.395) | 0.111 |
| Cardiogenic shock | 1.655 (1.162 - 2.356) | 0.005 | 1.523 (1.062 - 2.183) | 0.022 |
| Hypertension | 1.316 (1.102 - 1.572) | 0.002 | 1.029 (0.850 - 1.244) | 0.771 |
| Dyslipidemia | 1.225 (0.958 - 1.566) | 0.106 | 1.195 (0.932 - 1.532) | 0.161 |
| Current smokers | 1.190 (0.993 - 1.425) | 0.059 | 1.157 (0.936 - 1.429) | 0.177 |
| NT-ProBNP | 1.001 (1.001 - 1.002) | <0.001 | 1.000 (1.000 - 1.001) | 0.205 |
| ACEIs | 1.452 (1.217 - 1.731) | <0.001 | 1.282 (1.068 - 1.539) | 0.008 |
| Atorvastatin | 1.105 (0.824 - 1.398) | 0.305 | 1.015 (0.748 - 1.376) | 0.924 |
| Rosuvastatin | 1.215 (1.005 - 1.469) | 0.044 | 1.213 (0.877 - 1.677) | 0.244 |
| Simvastatin | 1.819 (1.186 - 2.789) | 0.006 | 1.939 (1.172 - 3.208) | 0.005 |
| eGFR < 60mL/min/1.73m^2^ | 1.778 (1.457 - 2.170) | <0.001 | 1.356 (1.091 - 1.686) | 0.006 |
| ACC/AHA type B2 lesion | 1.124 (0.936 - 1.350) | 0.211 | 1.158 (0.962 - 1.395) | 0.121 |
| Multivessel disease | 1.923 (1.593 - 2.322) | <0.001 | 1.825 (1.488 - 2.237) | <0.001 |
| Stent diameter < 3.0mm | 1.135 (0.941 - 1.369) | 0.184 | 1.000 (0.825 - 1.212) | 0.998 |
| Number of stent | 1.122 (1.012 - 1.245) | 0.029 | 0.945 (0.842 - 1.061) | 0.339 |

MACE: major adverse cardiac events; HR: hazard ratio; CI: confidence interval; Group A1: high-intensity statin/normoglycemia; Group A2: high-intensity statin/prediabetes; Group A3: high-intensity statin/T2DM; LVEF: left ventricular ejection fraction; STEMI: ST-segment elevation myocardial infarction; NT-ProBNP: N-terminal pro-brain natriuretic peptide; ACEI: angiotensin converting enzyme inhibitors; eGFR: estimated glomerular filtration rate; ACC/AHA: American College of Cardiology/American Heart Association.
